# Apoptotic bodies inhibit inflammation by PDL1–PD1‐mediated macrophage metabolic reprogramming

**DOI:** 10.1111/cpr.13531

**Published:** 2023-08-08

**Authors:** Tao Jiang, Yanmin Xia, Wenzhe Wang, Jinbo Zhao, Wenhao Liu, Shiyu Liu, Songtao Shi, Bei Li, Xiaoning He, Yan Jin

**Affiliations:** ^1^ Department of Thoracic Surgery, Tangdu Hospital Fourth Military Medical University Xi'an China; ^2^ State Key Laboratory of Oral & Maxillofacial Reconstruction and Regeneration, National Clinical Research Center for Oral Diseases, Shaanxi International Joint Research Center for Oral Diseases, Center for Tissue Engineering, School of Stomatology The Fourth Military Medical University Xi'an China; ^3^ South China Center of Craniofacial Stem Cell Research, Guanghua School of Stomatology Sun Yat‐sen University Guangzhou China

## Abstract

Apoptosis triggers immunoregulation to prevent and suppress inflammation and autoimmunity. However, the mechanism by which apoptotic cells modulate immune responses remains largely elusive. In the context of allogeneic mesenchymal stem cells (MSCs) transplantation, long‐term immunoregulation is observed in the host despite the short survive of the injected MSCs. In this study, utilizing a mouse model of acute lung injury (ALI), we demonstrate that apoptotic bodies (ABs) released by transplanted human umbilical cord MSCs (UC‐MSCs) convert the macrophages from a pro‐inflammatory to an anti‐inflammatory state, thereby ameliorating the disease. Mechanistically, we identify the expression of programmed cell death 1 ligand 1 (PDL1) on the membrane of UC‐MSCs‐derived ABs, which interacts with programmed cell death protein 1 (PD1) on host macrophages. This interaction leads to the reprogramming of macrophage metabolism, shifting from glycolysis to mitochondrial oxidative phosphorylation via the Erk‐dependent pathway in ALI. Importantly, we have reproduced the PDL1–PD1 effects of ABs on metabolic switch using alveolar macrophages from patients with ALI, suggesting the potential clinical implications of developing therapeutic strategies for the patients.

## Graphical abstract

Programmed cell death 1 ligand 1 on antibodies reprogramed the metabolic process of macrophages by switching the glycolysis to mitochondrial oxidative phosphorylation through Erk dependent pathway.

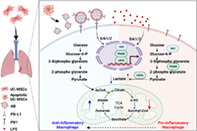

## 1 INTRODUCTION

Apoptosis, as a significant biological process, plays a critical role in development, tissue homeostasis, regeneration, and particularly inflammation.^1^, ^2^, ^3^, ^4^ Timely apoptosis and efficient cell clearance are essential to restrict infection, alleviate inflammation, and maintain immune tolerance against self‐antigens.^5^, ^6^ However, the mechanism by which apoptotic cells regulate immune responses remains incompletely understood. Intriguingly, mesenchymal stem cells (MSCs) have demonstrated clinical efficacy in treating various inflammatory diseases.^7^, ^8^, ^9^, ^10^ Notably, transplanted MSCs undergo apoptosis, characterized by cell rounding and shrinking, chromatin condensation, and the formation of plasma membrane blebs or apoptotic bodies (ABs).^11^, ^12^ Our previous studies have shown that ABs released by MSCs can be phagocytosed by liver macrophages, resulting in an anti‐inflammatory effect.^13^, ^14^ Nevertheless, the detailed mechanisms underlying the functional impact of ABs derived from MSCs on macrophages, particularly in the context of inflammation, remain largely unknown.

Under the inflammatory conditions, such as acute lung injury (ALI), characterized by severe protein‐rich pulmonary edema and hypoxaemia,^15^ macrophages serve as the primary responders among immune cells, playing a crucial role in the initiation and progression of inflammation.^16^, ^17^, ^18^ Moreover, macrophages are widely recognized as the primary ‘professional’ phagocytes responsible for the clearance of apoptotic cells.^19^ During inflammation, macrophages undergo metabolic reprogramming, shifting their core metabolism from oxidative phosphorylation (OXPHOS) to glycolysis, upon activation by lipopolysaccharide (LPS), a Gram‐negative bacterial product.^20^, ^21^ This metabolic shift in macrophages leads to increased secretion of pro‐inflammatory cytokines.^20^, ^22^, ^23^ Notably, the metabolic processes that induce glycolytic activation in pro‐inflammatory macrophages, triggered by the Toll‐like receptor 4 agonist LPS, are downregulated in anti‐inflammatory macrophages.^24^, ^25^ Building upon our previous findings demonstrating the anti‐inflammation effects of macrophages following the phagocytosis of ABs derived from MSCs, we hypothesized that ABs from MSCs might regulate the metabolism of macrophages.

The programmed cell death 1 ligand 1‐programmed cell death protein 1 (PDL1–PD1) immune checkpoint pathway has been recognized for its role in preventing excessive tissue damage during inflammatory states.^26^ PDL1 is expressed on various cell types in the body, including MSCs and its expression increases in the presence of inflammation.^26^, ^27^ In addition, exosomal PDL1 binds to PD1 on T cells, acting as an immunosuppressant that promotes wound healing.^28^ Recent studies have reported that the PDL1–PD1 axis regulates the glycolytic metabolism of T lymphocytes during inflammation.^29^, ^30^, ^31^ However, the expression of PDL1 on ABs and the effects of PDL1–PD1 signalling on macrophage metabolism during inflammation remain unknown.

In this study, we aimed to investigate the anti‐inflammatory effect of ABs derived from umbilical cord MSCs (UC‐MSCs) in an ALI model and the metabolic regulation of ABs in macrophages. We found that UC‐MSCs underwent apoptosis following intratracheal transplantation in ALI mice. The therapeutic effect of UC‐MSCs on ALI was found to rely on the presence of ABs, which induced a transition of macrophages from a pro‐inflammatory state to an anti‐inflammatory state. Furthermore, UC‐MSCs‐derived ABs expressed PDL1, and ABs drove the metabolic reprogramming of macrophage by inhibiting glycolysis through the PDL1–PD1 signalling. This metabolic shift promoted the anti‐inflammatory properties of macrophages and mitigated the severity of ALI. Our findings reveal that metabolic reprogramming of macrophages by MSC‐derived ABs to switch macrophages into anti‐inflammatory state holds potent therapeutic potential in inflammatory diseases.

## 2 MATERIALS AND METHODS

### 2.1 Animal care

8‐ to 10‐week‐old male C57BL/6J mice for ALI model were purchased from Animal Center of the Fourth Military Medical University, China. Female DBA/1 mice (6–8 weeks old) for inflammatory arthritis model were obtained from Beijing Vital River Laboratory Animal Technology Co., Ltd. The animals were maintained under pathogen‐free conditions with constant temperature, humidity, and 12:12‐h light–dark cycle, and with food and water easily accessible. All animal experiments were performed following the guidelines set by the Animal Care Committee of the Fourth Military Medical University, Xi'an, China.

### 2.2 Animal models of ALI

ALI model was induced in 8–10 weeks old C57BL/6J mice by intratracheal administration of LPS (Sigma, 5 mg/kg in 50 μL saline). Control mice received 50 μL saline intratracheally. After 4 h, ABs or PKH67 labelled ABs, or ABs + anti‐PD1 were intratracheal administered (10 μg ABs in 50 μL saline per mouse). The severity of lung tissue damage and inflammatory cell infiltration in ALI mice was individually scored using a previously described scoring system.^32^ The total score per mouse was obtained by adding up the scores (ranging from 0 to 4) for four assessment items. Cytokine concentrations were measured on cell‐free bronchoalveolar lavage fluid (BALF) using the enzyme‐linked immunosorbent assay kits (Meimian, Jiangsu, China) following the manufacturer's instructions.

### 2.3 Isolation and characterization of Abs

UC‐MSCs were isolated from umbilical cord of healthy puerperal women as previously described.^33^ Cells were cultured in basal culture medium containing α‐MEM medium (Gibco), 10% FBS (Sijiqing, Zhejiang, China), 2 mM L‐glutamine (Invitrogen Life Technology, Carlsbad, California), 100 U/mL penicillin and 100 U/mL streptomycin (Invitrogen). According to the corresponding experiments, UC‐MSCs were pre‐labelled with PKH67 or treated with staurosporine (STS) at a concentration of 0.2 μM to produce ABs. UC‐MSCs were used at Passages 4–8 in the following study.

ABs were isolated from the culture supernatant of UC‐MSCs undergoing induced apoptotic using gradient centrifugation, following a previously established method.^13^ Briefly, UC‐MSCs were treated with 0.5 μM STS for 12–16 h to induce apoptosis. The culture supernatant was subjected to additional centrifuged at 800 × g for 10 min to remove large debris. The supernatant was additional centrifugation at 16,000 × g for 30 min at 4°C, and the resulting pellet was washed twice with PBS and resuspended in PBS for further use. The samples were observed using a scanning electron microscope (S4800, Hitachi, Japan). Moreover, the size distribution of ABs was measured by nanoparticle tracking analysis (NTA; Ribobio Ltd., Guangzhou, China). The ABs marker caspase3 was analysed by western blot. ABs were also stained with Annexin‐5 (Roche, Basel, Switzerland), and observed using laser scanning confocal microscope (FV1000, Olympus, Japan).

### 2.4 Primary bone marrow‐derived macrophages culture

Primary bone marrow‐derived macrophages (BMDMs) were isolated from the femurs and tibiae bone marrow of CIA or C57BL/6J mice. The cells were cultured in RPMI‐1640 medium with 50 ng/mL recombinant macrophage colony‐stimulating factor (PeproTech, Rocky Hill, New Jersey) for a period of 5–7 days. Then, naive macrophages were collected and stimulated with 200 ng/mL LPS (Sigma‐Aldrich, St. Louis., Missouri) to generate pro‐inflammatory macrophages for 24 h.

### 2.5 Human alveolar macrophages culture

Primary alveolar macrophages were isolated from the lungs of mice or patients (Tangdu Hospital, Xian, China). This was achieved by bronchoalveolar lavage with five washes of normal saline at room temperature. Cells were pelleted and seeded in plates with complete RPMI‐1640 medium. Alternatively, the cells were stained with antibodies for flow cytometric analysis (Cytoflex, Beckman Coulter, California). The collection of human alveolar macrophages was performed with the approval and oversight of the Tangdu Hospital ethics committee (202103‐121). Written informed consent was obtained from all patients involved.

### 2.6 Flow cytometry

To assess the transition of macrophages from pro‐inflammatory to anti‐inflammatory states upon treatment with various agents, CD64 and CD80 (Biolegend, San Diego, California) were chosen as markers of pro‐inflammatory macrophages, and CD206 (Biolegend, San Diego, California) as a marker of the anti‐inflammatory macrophages. The markers were measured using flow cytometric analysis (Cytoflex, Beckman Coulter, California).

### 2.7 Immunofluorescence staining

Cells were fixed in 4% paraformaldehyde‐PBS for 15 min. After washing with PBS[AUTHOR: Please define (2‐NBDG, BCA, qRT‐PCR, FCCP, PBS, IgG, TNF‐α, IL, KEGG), the cells were permeabilized with 0.1% Triton X‐100 in PBS for 20 min at room temperature. Then cells were blocked in 10% normal goat serum and incubated with antibody overnight at 4°C. After washed in PBS again, cells were incubated with Alexa Fluor 488‐labelled secondary antibody for 1 h at room temperature. In addition, 1 mg/mL of 4,6‐diamino‐2‐phenyl indole (DAPI) in PBS were used to stain the nuclei. Fluorescence images were captured with a laser scanning confocal microscope (FV1000, Olympus, Japan).

For in vivo studies, the lungs were harvested from sacrificed mice and fixed overnight with 4% paraformaldehyde, then cryoprotected with 30% sucrose and embedded in the optimal cutting temperature compound. The frozen sections were cut at 10 μm thick (CM1950, Leica, Solms, Germany) and stained with primary antibodies anti‐F4/80 and anti‐Ly6G (abcam, Cambridge, UK) overnight at 4°C. The sections were then stained by Alexa Fluor 568 goat anti‐rabbit IgG for 30 min at room temperature at concentrations of 1:300 and counterstained with DAPI.

For intracellular mitochondria staining, the Mito‐Tracker Deep Red (Thermo Fisher Scientific, Massachusetts) was used. Cells were cultured in confocal small dish and supplemented with 100 nM Mito Tracker Deep Red at 37°C for 30 min, followed by incubation with Hoechst for 5 min. Then Fluorescence images were obtained by confocal fluorescence microscope (FV1000, Olympus, Japan).

### 2.8 RNA‐sequencing

For RNA‐sequencing analysis, RAW264.7 cells were cultured and treated with LPS (200 ng/mL) alone or in combination with AB (1 μg/mL) or AB + anti‐PD1 (1 μM) for 24 h. Total RNA was isolated and used for RNA‐sequencing analysis. cDNA library construction and sequencing were performed by Beijing Genomics Institute using BGISEQ‐2000 platform. The sequencing data were filtered with SOAPnuke (v1.5.2), and the expression levels of genes were calculated by RSEM (v1.2.12), differential expression analysis was performed using the DESeq2 (v1.4.5)^34^ with *Q*‐value ≤ 0.05. To gain insights into the phenotypic changes, Gene Ontology (GO) analysis (http://www.geneontology.org/) and KEGG (https://www.kegg.jp/) pathway enrichment analysis of the annotated differentially expressed genes were performed by established procedures at BGI.

### 2.9 Seahorse metabolic flux analysis

Patients' alveolar macrophages or BMDMs (2 × 10^5^/well) were seeded in XF24‐cell culture plates (Seahorse Bioscience, USA). Pro‐inflammatory cells were treated with ABs or various reagents (anti‐PD1, PD0325901, AR234960) for 24 h. The oxygen consumption rate (OCR) and extra‐cellular acidification rate (ECAR) were measured in an XF24 Flux Analyser (Seahorse Bioscience, USA). In the OCR assay, cells were first measured under basal conditions, and then stimulated with oligomycin (1 μM), FCCP (1 μM), rotenone (1 μM), and antimycin A (1 μM) to determine different parameters of mitochondrial functions. In the ECAR assay, MitoPBN (40 μmoL/L) was pre‐treated for 1 h and then changed to the base medium and incubated at 37°C in a non‐CO_2_ incubator for 1 h. Glucose (10 mM), oligomycin (1 μM), and 2‐DG (50 mM) were subsequently added into the medium.

### 2.10 shRNA‐mediated knockdown of PD1 and PDL1 in BMDMs and ABs

For shRNA knockdown experiments, BMDMs and UC‐MSCs were treated with lentivirus, which respectively carried PD1 or PD‐L1 shRNAs, according to the manufacturers' protocol (Shanghai Genechem Co., Ltd). Briefly, BMDMs were infected with shPD1 lentivirus at a multiplicity of infection (MOI) of 100 and UC‐MSCs were infected with shPD‐L1 lentivirus at a MOI of 10. After 48 h of shRNA induction, the cells were analysed for expression levels of PD1 and PD‐L1 and used for subsequent experiments.

### 2.11 Statistical analyses

All experiments were repeated at least three times, and data are presented as mean ± SD. Statistical analysis was performed by Student's *t*‐test (twotailed) and oneway analysis of variance, with GraphPad Prism 8.0 (GraphPad Software, USA). The difference between groups was considered statistically significant for *p < 0.05, very significant for **p < 0.01, the most significant for ***p < 0.001. Graph analysis was performed using GraphPad Prism 8.0 (GraphPad Software, USA).

## 3 RESULTS

### 3.1 ABs released by UC‐MSCs prevent LPS‐induced inflammatory lung injury

Since the administration of human UC‐MSCs in mice prevented LPS‐induced ALI,^10^ we intratracheally injected human UC‐MSCs pre‐labelled with PKH67 to the ALI model in mice. Human UC‐MSCs were mainly found in lung tissue after 24 h transplantation (Figure S1A) and then disappeared after 48 h transplantation as observed by confocal microscope in vivo (Figure S1B). To determine whether the UC‐MSCs underwent apoptosis in the lung tissue, we performed TdT‐mediated dUTP Nick‐End Labeling (TUNEL) staining 24 h after transplantation. The UC‐MSCs, pre‐labelled with PKH67 (green), were examined for the presence of TUNEL‐positive cells (red), indicating apoptotic UC‐MSCs in the injected lung tissue (Figure S1C).

**FIGURE 1:**
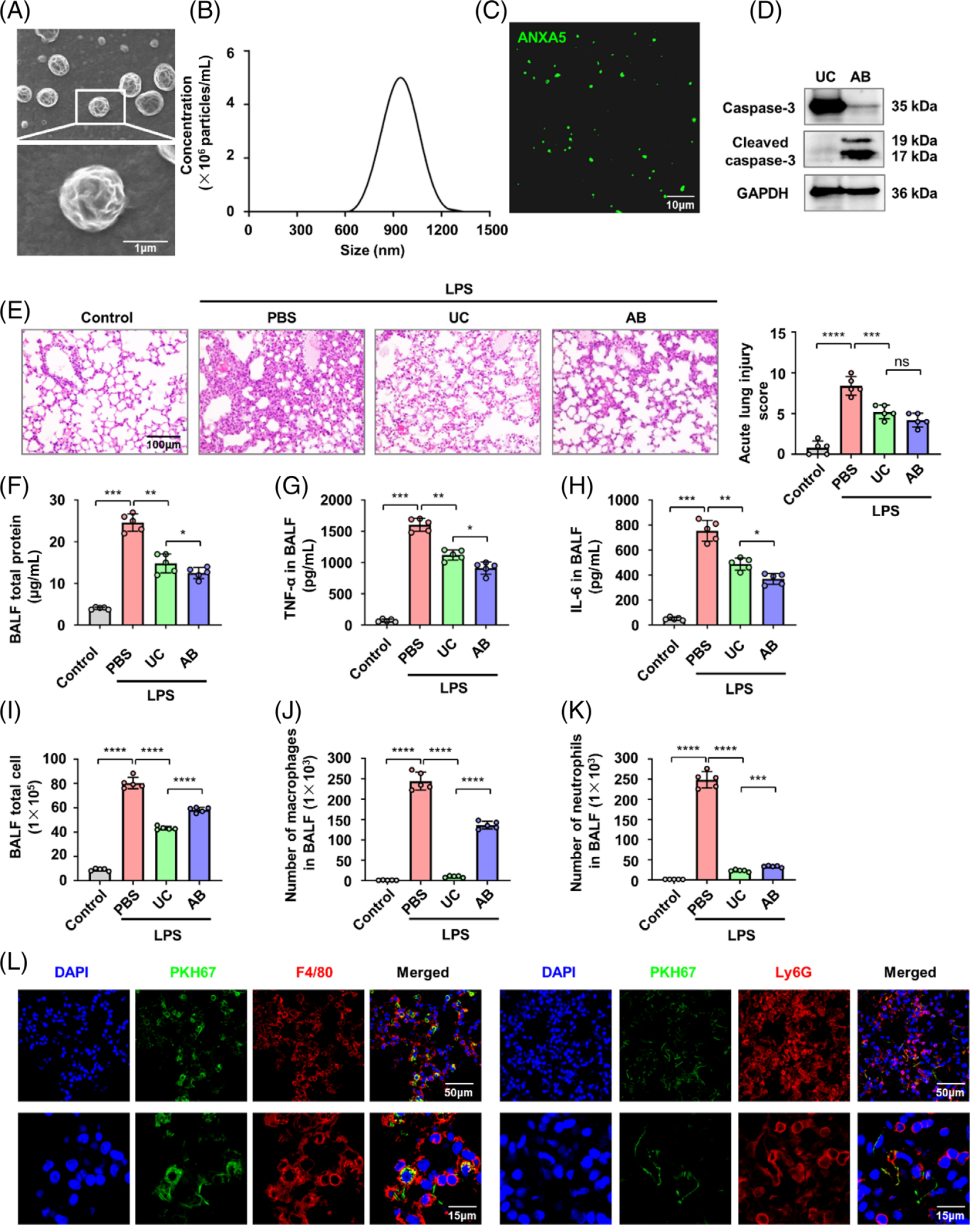
Apoptotic bodies released by umbilical cord mesenchymal stem cells (UC‐MSCs) prevent lipopolysaccharide (LPS)‐induced inflammatory lung injury. (A) Morphology of UC‐MSCs‐derived antibodies (ABs) shown by scanning electron microscope. Scale bar, 1 μm. (B) Size distribution of ABs measured by nanoparticle tracking analysis. (C) Confocal microscopy images of ABs stained with ANXA5/annexin V showing phosphatidylserine exposed on the surface. Scale bar, 10 μm. (D) Western blots of the apoptotic marker protein caspase 3 and cleaved‐caspase 3 for UC‐MSCs‐derived ABs. (E) haematoxylin and eosin staining of lung tissue sections (left) and quantification of the severity of lung tissue damage and inflammatory cell infiltration (right) showed UC‐MSCs and ABs derived from US‐MSCs both suppressed lung inflammation of LPS‐induced acute lung injury (ALI; *n* = 5). Scale bar, 100 μm. (F) Total protein in bronchoalveolar lavage fluid (BALF) was measured by BCA assay (*n* = 5). (G and H) ELISA for TNF‐α (G) and IL‐6 (H) in BALF (*n* = 5). (I–K) Cell infiltration of lung was measured in BALF by flow cytometric analysis, total cells (I), macrophages (J), and neutrophils (K) (*n* = 5). (L) Immunofluorescence staining of ABs (Green) and F4/80 or Ly6G (Red) in the lungs after intratracheal administration of ABs. Scale bars, 50 and 15 μm. *p < 0.05; **p < 0.01; ***p < 0.001. Error bars are mean ± SD.

Next, UC‐MSCs were pre‐treated with STS to induce apoptosis and ABs were isolated from apoptotic UC‐MSCs.^13^ The morphology and size distribution of the ABs were characterized by scanning electron microscopy and NTA (Figure 1A, B). The ABs typically displayed a round shape (Figure 1A) and composed a homogenous population with sizes ranging from 600 to 1200 nm in diameter (Figure 1B). Then, we determined ABs by positive ANXA5 (annexin A5) staining (Figure 1C). Western blot analysis showed that the isolated ABs expressed the specific marker caspase 3 (Figure 1D). To evaluate the therapeutic effects of UC‐MSCs and ABs on ALI, lung inflammation was assessed by haematoxylin and eosin staining of lung tissue section. The numbers of total cells, neutrophils, and macrophages, as well as the levels of total protein, TNF‐α, and IL‐6 in BALF were measured. Both UC‐MSCs and ABs derived from US‐MSCs significantly suppressed lung inflammation in the LPS‐induced ALI model (Figures 1E–K and S1D). To investigate the role of ABs in mediating the therapeutic effects of UC‐MSCs, the release of ABs was inhibited using sertraline, and the therapeutic effect of UC‐MSCs was examined (Figure S1E). We showed that inhibition of ABs release compromised the alleviation of lung inflammation by UC‐MSCs (Figure S1F–I), suggesting that the released ABs play a crucial role in the therapeutic effects. To further explore which type of immune cells recognized ABs in inflammatory lung tissue, F4/80 positive macrophages (Red) and Ly6G positive neutrophiles (Red) were stained after administration of ABs pre‐labelled with PKH67 (Green). The results showed that F4/80 positive macrophages were responsible for phagocytosing the ABs (Figure 1L). The data indicated that ABs released from apoptotic UC‐MSCs exert therapeutic effects on ALI by suppressing lung inflammation, primarily through their recognition and uptake by macrophages.

### 3.2 ABs inhibit pro‐inflammation of macrophages through PDL1–PD1 pathway

**FIGURE 2:**
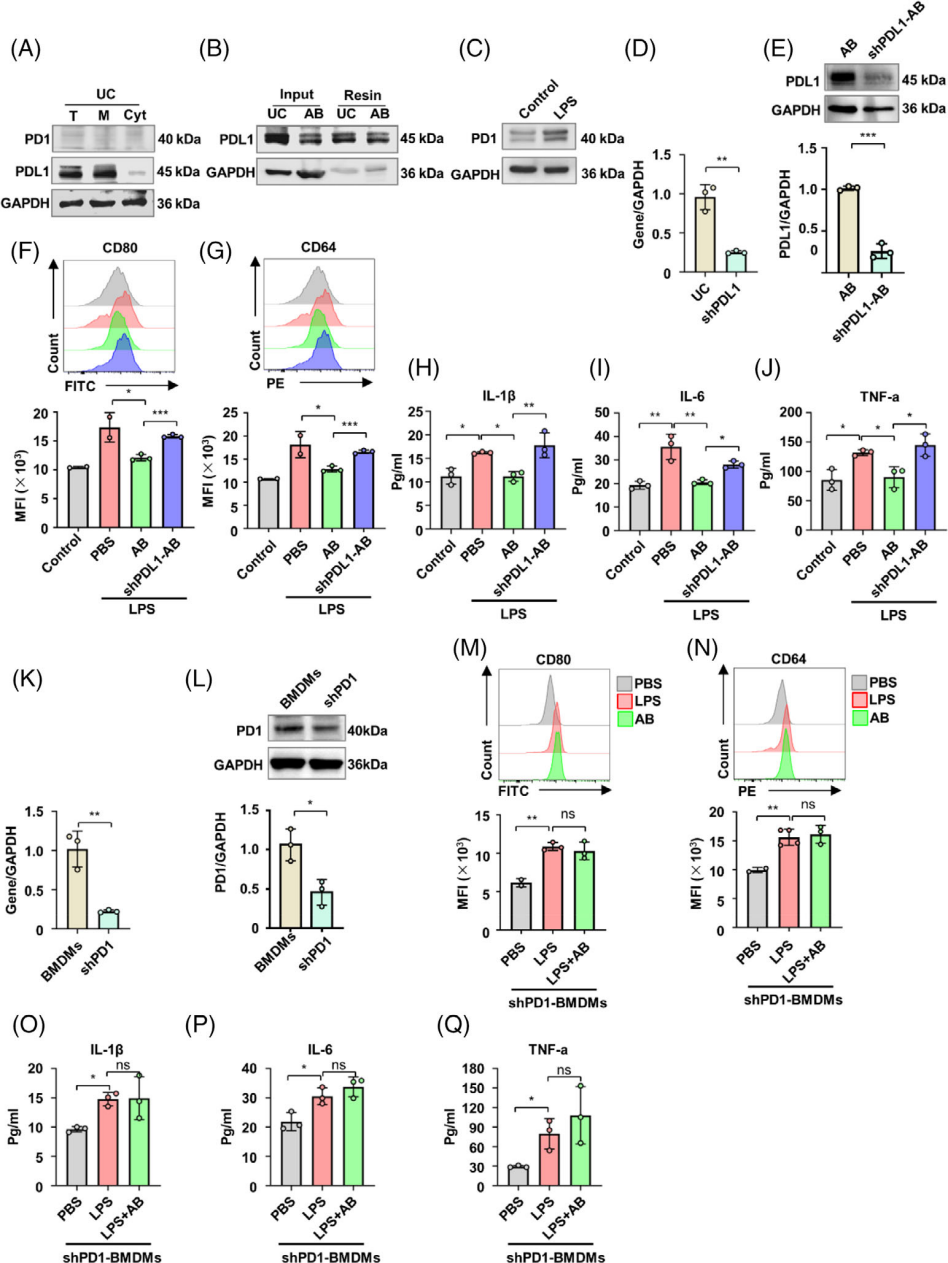
Antibodies (ABs) inhibit pro‐inflammation of macrophages through programmed cell death 1 ligand 1–programmed cell death protein 1 (PDL1–PD1) pathway. (A) The expressions of PD1 and PDL1 were assessed by western blot analysis. Left lane, total protein, middle lane, membrane protein, right lane, cytoplasm protein. PDL1 was mainly expressed on plasma membrane of human umbilical cord mesenchymal stem cells (UC‐MSCs). (B) Cell surface biotinylation assay of PDL1 on ABs. Right two lanes, western blot for PDL1 following neutravidin pull down from UC‐MSCs and ABs. Left two lanes, input, not biotinylated cells. (C) The expression of PD1 on the alveolar macrophage of mice was increased after stimulated by lipopolysaccharide (LPS). (D) PDL1 expression of UC was detected by qRT‐PCR analysis (*n* = 3). (E) Western blot analysis (top) and quantitative analysis of PDL1 protein expression (bottom) showed that PDL1 expression of ABs was decreased after transfected with shPDL1 (*n* = 3). (F,G) The expression of pro‐inflammation surface markers CD80 and CD64 of bone marrow‐derived macrophages (BMDMs) in different groups were analysed by flow cytometry (*n* = 3). Representative flow cytometry histogram of macrophages (top). Quantifications of CD80 and CD64 were presented with mean fluorescence intensity (MFI) (bottom). (H–J) Enzyme‐linked immunosorbent assay (ELISA) for IL‐1β (H), IL‐6 (I), and TNF‐α (J) in supernatant of BMDMs in different groups. (K) PD1 expression of BMDMs was detected by qRT‐PCR analysis (*n* = 3). (L) Western blot analysis (top) and quantitative analysis of PD1 protein expression (bottom) showed that PD1 expression of BMDMs was decreased after transfected with shPD1. (M,N) The expressions of CD80 and CD64 of shPD1‐BMDMs in different groups were analysed by flow cytometry (*n* = 3). Representative flow cytometry histogram of shPD1 macrophages (top). Quantifications of CD80 and CD64 were presented with MFI (bottom). (O–Q) ELISA for IL‐1β (O), IL‐6 (P), and TNF‐α (Q) in supernatant of shPD1‐BMDMs (*n* = 3). ns, not significant. *p < 0.05; **p < 0.01; ***p < 0.001. Error bars are mean ± SD.

Recent study showed that exosomal PDL1 regulated the inflammatory response to maintain proper immune homoeostasis in wound healing.^28^ We next sought to unveil whether PDL1 was also expressed by ABs, which are derived from plasma membrane outward blebbing when cells are in the late stage of apoptosis.^35^ We found that PDL1, but not PD1 was expressed on the plasma membrane of human UC‐MSCs (Figure 2A). Moreover, we labelled the surface molecules with biotin and used neutravidin ultralink resin beads to capture biotinylated surface proteins. Western blot analysis was then applied to show expression level of captured proteins by anti‐PDL1 antibodies. The results showed the expression of PDL1 both on the cell surface of UC‐MSCs and on the membrane of ABs (Figure 2B). On the other hand, the expression of PD1 on alveolar macrophage in mice was increased after stimulation with LPS from *E. coli* O111:B4 (Figure 2C). Moreover, we detected the PD1 expressions in interstitial macrophages and monocyte‐derived macrophages of ALI mice. The results showed that the expression of PD1 in macrophage population in the inflammatory lung was also increased (Figure S2A,B). Macrophages were reported to be polarized to pro‐inflammatory state and to release pro‐inflammatory cytokines by LPS, but reversed to anti‐inflammatory state by MSCs in ALI.^36^ To know whether ABs regulate the polarization state of macrophages through PDL1–PD1 axis, we stimulated bone marrow‐derived macrophages (BMDMs) with LPS and studied the effect of ABs on BMDMs. We showed that ABs decreased the expression of pro‐inflammatory makers (CD80 and CD64) and production of pro‐inflammatory cytokines, including IL‐1β, IL‐6, and TNF‐α in BMDMs stimulated by LPS (Figure 2F–J). Functionally, inhibition of PDL1 expression in UC‐MSCs by shRNA downregulated the expression of PDL1 on ABs (Figure 2D,E). shPDL1‐ABs treatment increased the expression of pro‐inflammatory makers and cytokines in LPS‐stimulated BMDMs (Figure 2F–J). Additionally, when the PDL1 is blocked by the anti‐PDL1 antibody, the effects of ABs on macrophage polarization are abolished, as indicated by the expression levels of the genes related to pro‐inflammatory (IL‐1β, IL‐6, TNF‐α, and NOS2) and anti‐inflammatory (Arg1 and IL‐10) features of macrophages (Figure S2C–H). On the other hand, ABs were unable to inhibit the expression of pro‐inflammatory makers and cytokines production in LPS‐stimulated BMDMs with PD1 knockdown (Figure 2K–Q). These data suggest that ABs inhibit pro‐inflammatory polarization and cytokines production of macrophages through the interaction between PDL1 on ABs and PD1 on macrophages.

### 3.3 Blockade of PDL1–PD1 attenuates the therapeutic effect of ABs on ALI

**FIGURE 3:**
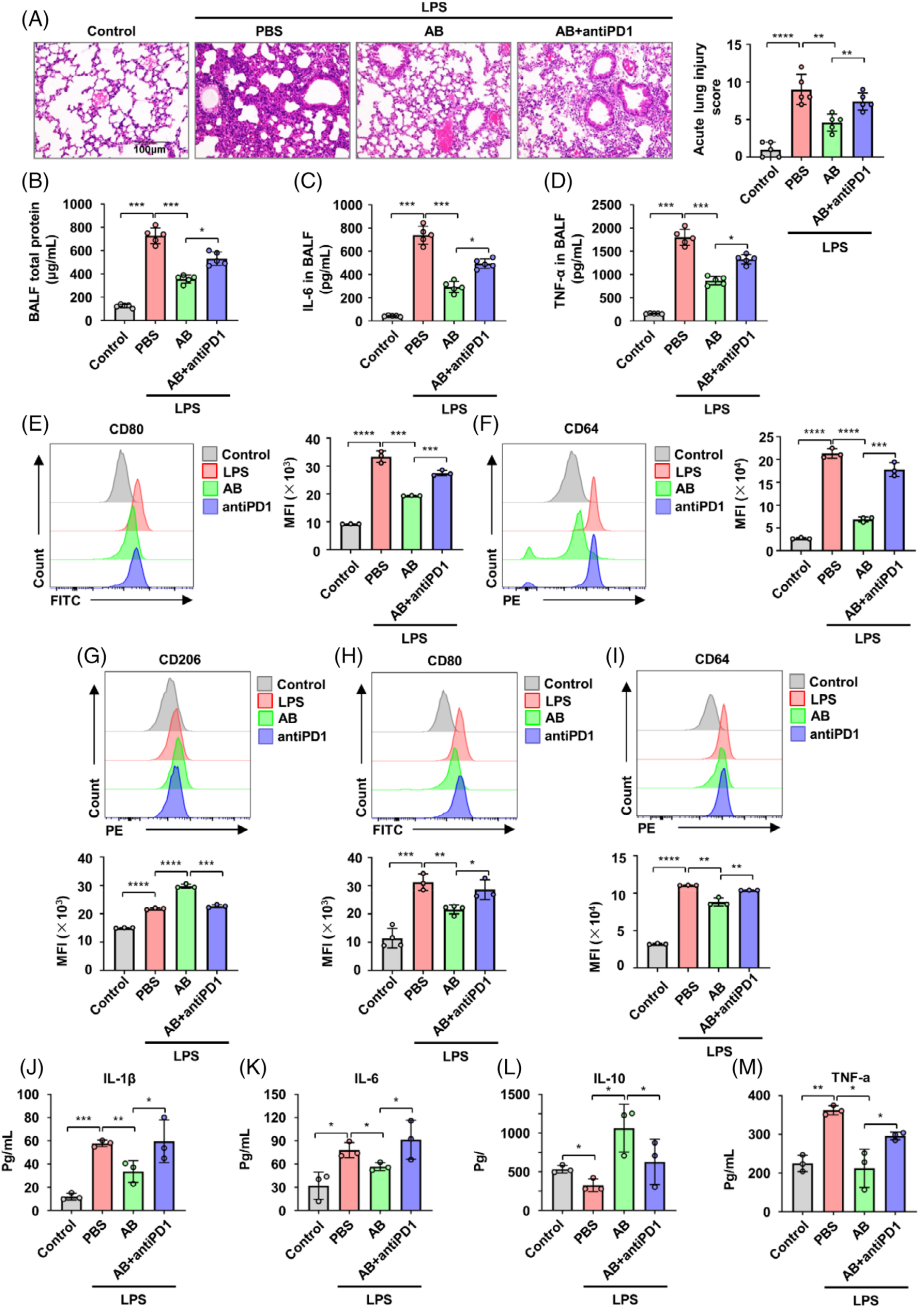
Blockade of programmed cell death 1 ligand 1–programmed cell death protein 1 (PDL1–PD1) attenuates the therapeutic effect of antibodies (ABs) on acute lung injury (ALI). (A) Haematoxylin and eosin (H&E) staining of lung tissue sections (left) and quantification of the severity of lung tissue damage and inflammatory cell infiltration (right) showed the therapeutic effect of ABs on ALI mice was attenuated after blockage of PDL1–PD1 interaction with anti‐PD1 antibody (*n* = 5). Scale bar, 100 μm. (B) Total protein in bronchoalveolar lavage fluid (BALF) was measured by BCA assay (*n* = 5). (C,D) enzyme‐linked immunosorbent assay (ELISA) for IL‐6 (C) and TNF‐α (D) in BAL fluid (*n* = 5). (E) Representative flow cytometry histogram and quantification of CD80 expression in alveolar macrophages from control and acute lung injury (ALI) mice (*n* = 3). (F) Representative flow cytometric analysis and quantification of CD64 expression in alveolar macrophages from control and ALI mice (*n* = 3). (G) Representative flow cytometry histogram and quantification of anti‐inflammatory marker CD206 expression in bone marrow‐derived macrophages (BMDMs) and BMDMs treated with lipopolysaccharide (LPS), ABs or ABs + anti‐PD1 (*n* = 3). (H,I) Representative flow cytometry histograms and quantification of pro‐inflammatory markers CD80 (H) and CD64 (I) expressions in BMDMs and BMDMs treated with LPS, ABs, or ABs + anti‐PD1 (*n* = 3). (J–M) ELISA for IL‐1β (J), IL‐6 (K), IL‐10 (L), and TNF‐α (M) in supernatant of BMDMs and BMDMs treated with LPS, ABs, or ABs + anti‐PD1 (*n* = 3). ns, not significant. *p < 0.05; **p < 0.01; ***p < 0.001. Error bars are mean ± SD.

We next aimed at deciphering whether the therapeutic effect of ABs on ALI depends on PDL1–PD1 axis. First, we detected the expression of PD1 in alveolar macrophages, interstitial macrophages, and monocyte‐derived macrophages of ALI mice treated with ABs and anti‐PD1 antibody. The results indicated that ABs and anti‐PD1 antibody treatment could reduce the expression of PD1 in macrophage population in inflammatory lung (Figure S3A–C). After blockage of PDL1–PD1 interaction with anti‐PD1 antibody, the therapeutic effect of ABs on ALI mice was attenuated (Figure 3A–D). The alveolar macrophages showed increased expression of pro‐inflammatory makers CD80 and CD64 in LPS‐induced ALI mice (Figure 3E,F). However, blockage of PDL1–PD1 axis by anti‐PD1 antibody reversed the effect of ABs on decreasing the expression of these markers in alveolar macrophages from ALI mice (Figure 3E,F). Consistently, we found that blockage of PDL1–PD1 by anti‐PD1 antibody also attenuated the therapeutic effect of ABs in type‐II collagen‐induced inflammatory arthritis. This was evidenced by increased lymphocyte infiltration, elevated expression of pro‐inflammatory cytokines (IL‐1β and TNF‐α) in articular cartilage, and higher levels of pro‐inflammatory cytokines in the serum (Figure S3D–H).

Based on the above observations, we next studied whether blocking PDL1–PD1 pathway switched the polarization state of macrophages. In vitro experiments were conducted using BMDMs treated with LPS and ABs in the presence or absence of an anti‐PD1 antibody. The results showed significantly increased expression of anti‐inflammatory marker CD206 and deceased expression of pro‐inflammatory makers CD80 and CD64 in BMDMs with supplementation of LPS and ABs compared with LPS alone (Figure 3G–I). However, when the PDL1–PD1 axis is blocked by the anti‐PD1 antibody, the effects of ABs on macrophage polarization are abolished, as indicated by the expression levels of CD206, CD80, and CD64 (Figure 3G–I). Anti‐PD1 antibody treatment also increased the expression of pro‐inflammatory factors (IL‐1β, IL‐6, and TNF‐α) and decreased the expression of the anti‐inflammatory factor IL‐10 in BMDMs treated with LPS and ABs (Figure 3J–M). Consistently, blocking the PDL1–PD1 axis by the anti‐PD1 antibody promotes pro‐inflammatory polarization in Raw264.7 cells stimulated with LPS and ABs, as evidenced by decreased expression of CD206 and increased expression of CD86 and MHC II (Figure S4A–C). Moreover, qRT‐PCR analysis showed that blocking PD1 in BMDMs treated with LPS and ABs leads to significant increases in the expressions of pro‐inflammatory cytokine genes, most notably *Il‐1β*, *Il‐6*, *Nos2*, and *TNF‐α*, while the expressions of anti‐inflammatory marker genes of *Arg1* and *Mrc1* were decreased (Figure S4D–I).

### 3.4 ABs contribute to the metabolic switch of macrophages through PDL1–PD1

**FIGURE 4:**
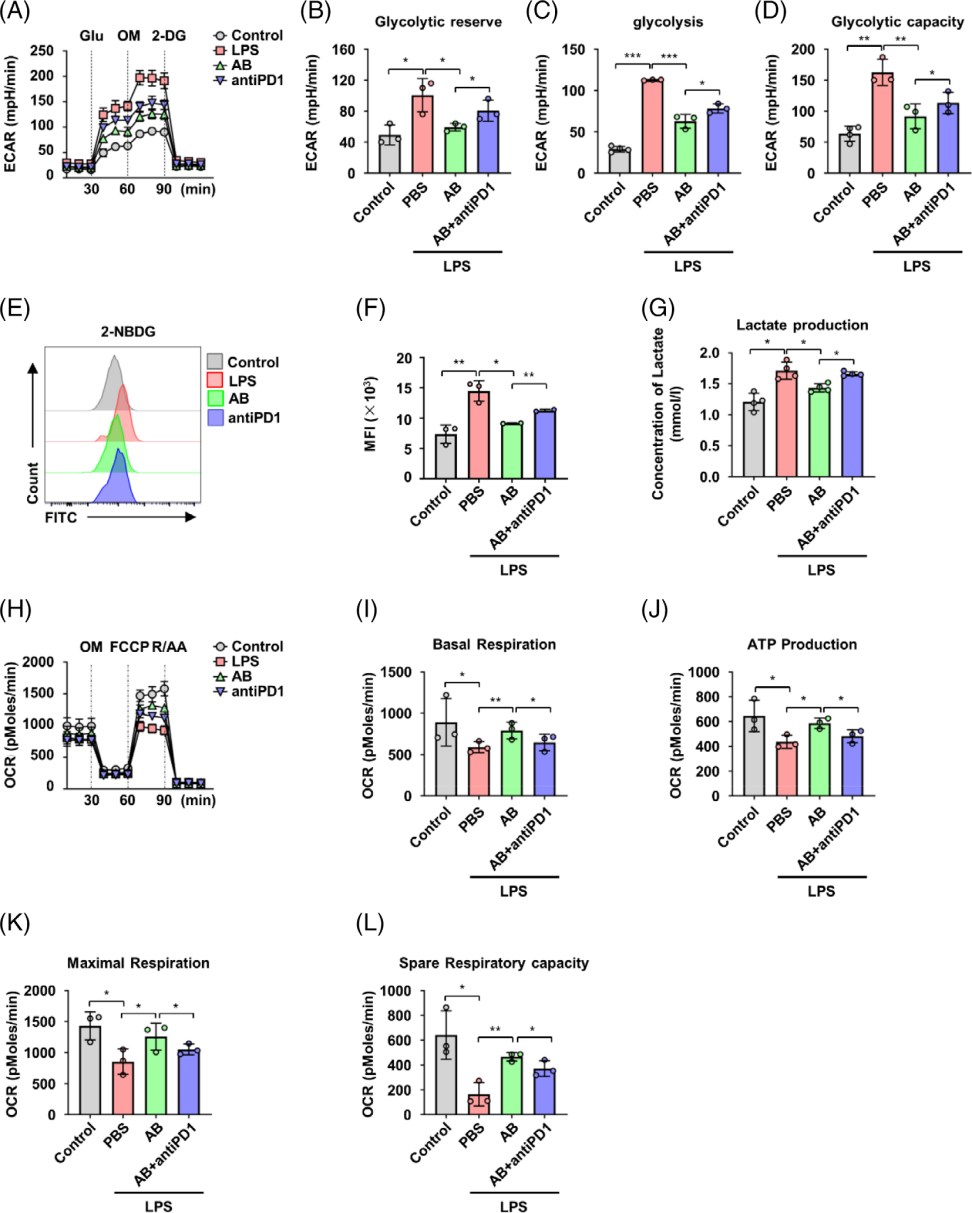
Antibodies (ABs) contribute to the metabolic switch of macrophages through programmed cell death 1 ligand 1–programmed cell death protein 1 (PDL1–PD1). (A) Line graph showing extra‐cellular acidification rate (ECAR) from seahorse analysis assessed in BMDMs and bone marrow‐derived macrophages (BMDMs) with supplementation of lipopolysaccharide (LPS), ABs or AB + anti‐PD1 (*n* = 3). (B–D) glycolytic reserve (B), glycolysis (C), and glycolytic capacity (D) in ECAR assay. (E) Representative flow cytometry histograms of 2‐NBDG staining. (F) Quantification of glucose uptake presented by 2‐NBDG staining (*n* = 3). (G) Lactate release in BMDMs and BMDMs with supplementation of LPS, ABs, or AB + anti‐PD1 (*n* = 4). (H) Line graph showing oxygen consumption rate (OCR) from seahorse analysis assessed in BMDMs and BMDMs with supplementation of LPS, ABs, or AB + anti‐PD1 (*n* = 3). (I–L) Basal respiration (I), ATP production (J), maximal respiration (K), and spare respiratory capacity (L) in OCR assay. ns, not significant. *p < 0.05; **p < 0.01; ***p < 0.001. Error bars are mean ± SD.

To gain insights into potential mechanisms through which ABs alter macrophage polarization, we performed global RNA sequencing (RNAseq) on Raw264.7 cells with treatment of LPS, LPS + ABs, or LPS + ABs + anti‐PD1 compared with untreated cells. The RNA‐seq analysis showed that the anti‐PD1 antibody significantly reversed the suppression of pro‐inflammatory cytokine and chemokine transcripts in LPS + ABs‐treated group (Figure S4J). As the RNA‐seq analysis showed that major metabolic pathways were affected by ABs, seahorse extracellular flux analysis, which measures OCR and ECAR, was performed on BMDMs and BMDMs with supplementation of LPS, LPS + ABs or LPS + ABs + anti‐PD1. The results showed that ABs suppressed the ECAR of BMDMs with supplementation of LPS, while LPS treatment elevated ECAR, consistent with inhibition of glycolysis (Figure 4A–D). However, the blockade of PDL1–PD1 pathway by anti‐PD1 antibody partially reversed the glycolysis inhibition in BMDMs treated with LPS and ABs. To validate the ECAR findings, we assessed BMDMs glucose uptake by flow cytometry using a fluorescence tagged, non‐metabolizable glucose analogue, 2‐NBD‐deoxyglucose, and found a decrease in glucose uptake in BMDMs treated with LPS and ABs compared with those treated with LPS alone. The anti‐PD1 antibody partially reversed this decrease in glucose uptake (Figure 4E,F). Lactate production by BMDMs was also reduced when supplemented with LPS and ABs, and this reduction was partially reversed by the anti‐PD1 antibody (Figure 4G). Concomitant with suppression of ECAR, ABs treatment induced elevation in OCR of BMDMs with LPS administration, indicating a metabolic switch to OXPHOS. The blockade of the PDL1–PD1 pathway partially curtailed the increase in OXPHOS and ATP production induced by ABs in LPS‐treated BMDM (Figure 4H–L). Previous study has demonstrated that there is intrinsic connection between structural organization and respiratory status of mitochondria.^37^ We thus conducted confocal fluorescence imaging studies to assess how ABs altered macrophage mitochondria morphology by overexpressing the fluorophore RFP (red fluorescent protein) specifically targeting to mitochondria. However, no significant changes in mitochondrial networks were observed in BMDMs with supplementation of LPS, LPS + ABs, or LPS + ABs + anti‐PD1 (Figure S5).

### 3.5 ABs reprogramme metabolic pathways of macrophages through Erk

**FIGURE 5:**
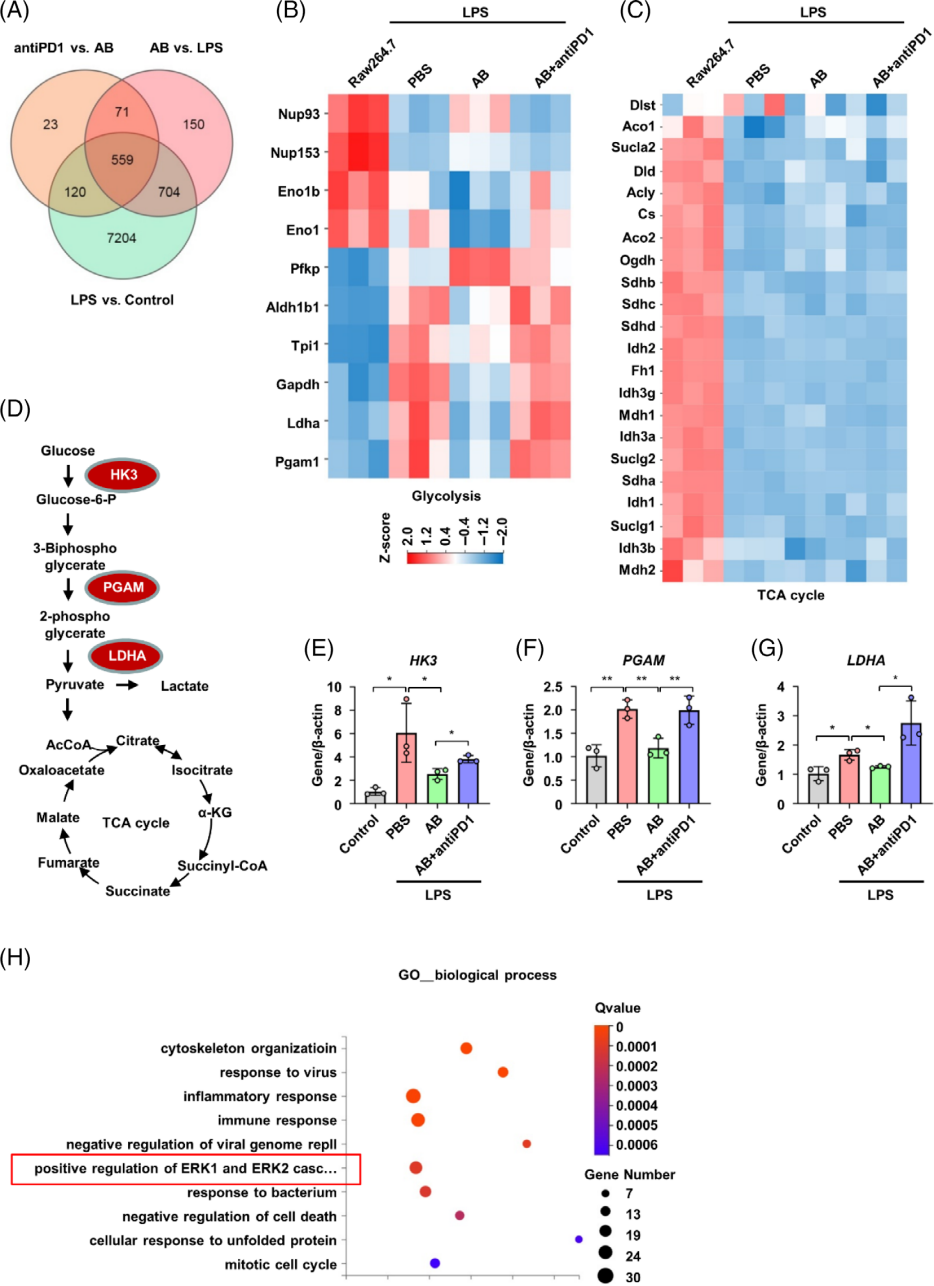
Antibodies (ABs) inhibit glycolytic pathway of macrophages through programmed cell death 1 ligand 1–programmed cell death protein 1 (PDL1–PD1). (A) Venn diagram depicts the number differentially expressed genes among three pairwise comparisons using principal component analysis. (B) Heatmap depicts differential gene expression for components of the glycolytic pathway. (C) Heatmap depicts differential gene expression for components of the tricarboxylic acid (TCA) cycle. Log2 fold‐change of pre‐mRNA splice variants and gene expression is shown. *p*‐values are adjusted for multiple testing. (D) Schematic depicting HK3, PGAM, and LDHA in integration of glycolysis and TCA cycle. (E–G) qRT‐PCR analysis of HK3 (E), PGAM (F), and LDHA (G) in bone marrow‐derived macrophages (BMDMs) and BMDMs with supplementation of lipopolysaccharide, ABs, or AB + anti‐PD1 (*n* = 3). (H) The correlated pathways were predicted downstream of PD1 in macrophages. ns, not significant. *p < 0.05; **p < 0.01. Error bars are mean ± SD. GO, Gene Ontology.

Since above data showed that ABs contributed to the metabolic switch of macrophages through PDL1–PD1 axis, the further detailed mechanism was explored. The Venn diagram shows differentially expressed genes among three pairwise comparisons using principal component analysis (Figure 5A). The RNA‐seq analysis showed that ABs downregulated most of the genes involved in the glycolysis, which were upregulated in macrophages after LPS treatment (Figure 5B). In contrast to glycolysis genes, most of the genes involved in tricarboxylic acid (TCA) cycle, a component of mitochondrial respiration, were unchanged or elevated after LPS + ABs treatment compared with LPS alone (Figure 5C). Moreover, RNA‐seq results also revealed most genes in fatty acid synthesis and fatty acid oxidation were not changed significantly after LPS + ABs treatment compared with LPS alone (Figure S6A,B). Notably, genes that control the three rate‐limiting steps of glycolysis including *Hk3*, *Pgam*, and *Ldha* (Figure 5D) were all upregulated in BMDMs treated with LPS alone, but downregulated by ABs, consistent with the notion that cells upregulated glycolysis to compensate for loss of mitochondrial OXPHOS (Figure 5E–G). However, anti‐PD1 antibody increased the expression of *Hk3*, *Pgam*, and *Ldha* in BMDMs treated with LPS and ABs (Figure 5E–G). In addition, the expression levels of *Gapdh*, *Tpl1*, and *Idh1* were not changed significantly among four groups (Figure S6C–E). These results indicate that ABs switch the glycolysis to OXPHOS in macrophages through PDL1–PD1 interaction, which contributes to the polarization of macrophages from pro‐inflammatory to anti‐inflammatory state.

**FIGURE 6:**
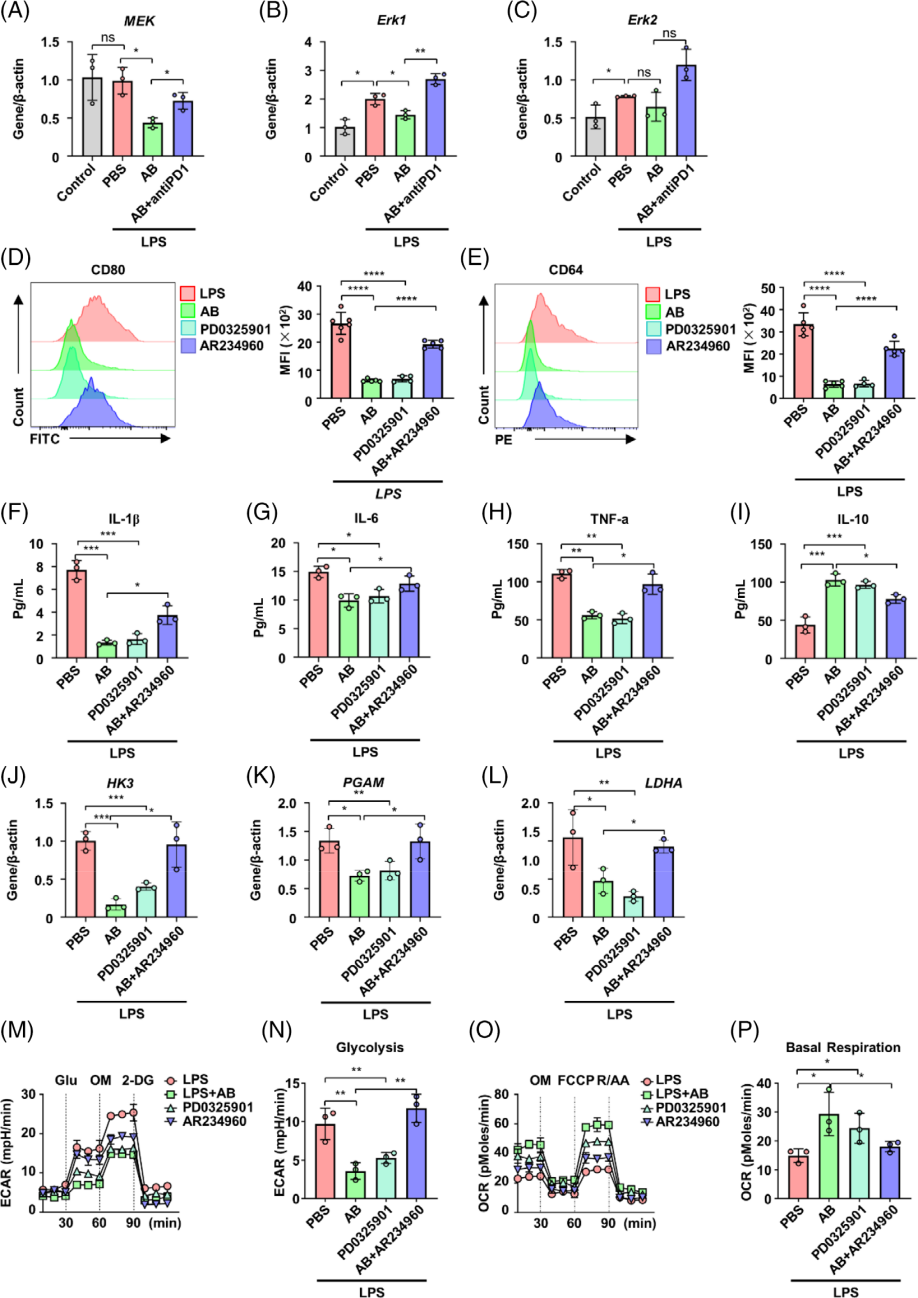
Antibodies (ABs) reprogramme metabolic pathways of macrophages through Erk. (A–C) qRT‐PCR analysis of MEK (A), Erk1 (B), and Erk2 (C) in bone marrow‐derived macrophages (BMDMs) and BMDMs with supplementation of LPS, ABs, or AB + anti‐PD1 (*n* = 3). (D) Representative flow cytometry histogram and quantification of CD80 expression in LPS‐induced BMDMs with supplementation of ABs or PD0325901 or AB + AR234960 (*n* = 5). (E) Representative flow cytometric analysis and quantification of CD64 expression in LPS‐induced BMDMs with supplementation of ABs or PD0325901 or AB + AR234960 (*n* = 5). (F–I) enzyme‐linked immunosorbent assay (ELISA) for IL‐1β (F), IL‐6 (G), TNF‐α (H), and IL‐10 (I) in supernatant of LPS induced with supplementation of ABs or PD0325901 or AB + AR234960 (*n* = 3). (J–L) qRT‐PCR analysis of HK3 (J), PGAM (K), and LDHA (L) in LPS‐induced BMDMs with supplementation of ABs or PD0325901 or AB + AR234960 (*n* = 3). (M) Line graph showing extra‐cellular acidification rate (ECAR) from seahorse analysis assessed in LPS‐induced BMDMs with supplementation of ABs or PD0325901 or AB + AR234960 (*n* = 3). (N) Effects of ERK pathway on glycolysis in ECAR assay. (O) Line graph showing OCR from seahorse analysis assessed in LPS‐induced BMDMs with supplementation of ABs or PD0325901 or AB + AR234960. (P) Effects of ERK pathway on basal respiration in OCR assay. ns, not significant. *p < 0.05; **p < 0.01; ***p < 0.001. Error bars are mean ± SD.

**FIGURE 7:**
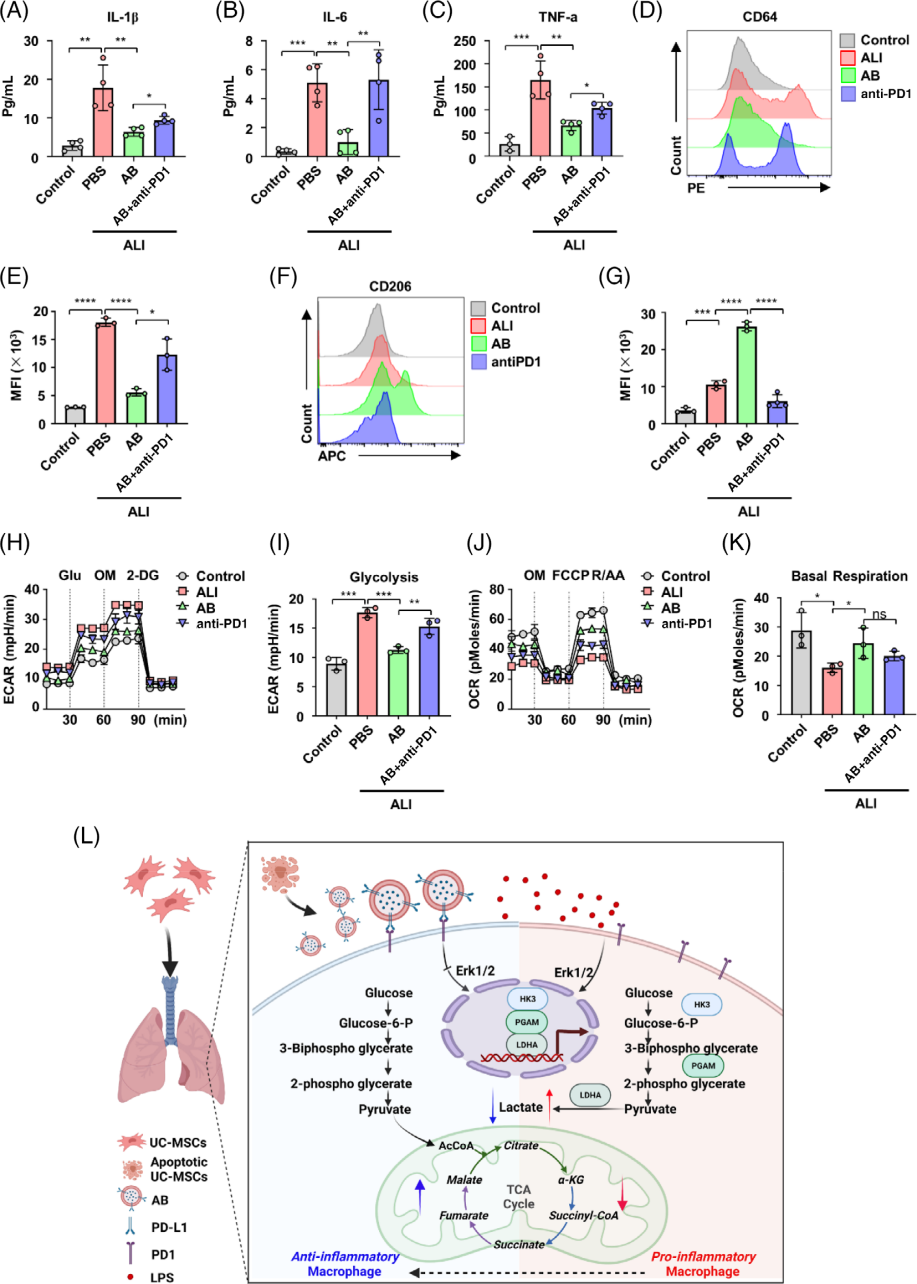
Antibodies (ABs) promote anti‐Inflammatory alveolar macrophages from acute lung injury (ALI) patients by programmed cell death 1 ligand 1–programmed cell death protein 1 (PDL1–PD1) pathway. (A–C) Enzyme‐linked immunosorbent assay (ELISA) for IL‐1β (A), IL‐6 (B), and TNF‐α (C) in supernatant of control and alveolar macrophages from ALI patients (*n* = 4). (D–E) Representative flow cytometry histogram (D) and quantification of CD64 expression (E) in control and alveolar macrophages from ALI patients (*n* = 3). (F,G) Representative flow cytometry histogram (F) and quantification of CD206 expression (G) in control and alveolar macrophages from ALI patients (*n* = 3). (H) Line graph showing extra‐cellular acidification rate (ECAR) from seahorse analysis assessed in alveolar macrophages from control and alveolar macrophages from ALI patients (*n* = 3). (I) Glycolysis in ECAR assay. (J) Line graph showing oxygen consumption rate (OCR) from seahorse analysis assessed in alveolar macrophages from control and alveolar macrophages from ALI patients (*n* = 3). (K) Basal respiration in OCR assay. (L) Graphic Summary shows the mechanisms of PDL1 on ABs reprogrammed the metabolic process of macrophages by switching the glycolysis to mitochondrial oxidative phosphorylation through Erk dependent pathway. ns, not significant. *p < 0.05; **p < 0.01; ***p < 0.001. Error bars are mean ± SD.

PI3K/Akt and MEK/Erk pathways are targets of PD1, and they play important roles in glucose metabolism.^38^, ^39^ Through GO pathway analysis and gene expression analysis, we found that Erk pathway was one of the most abundant signal transductions but not PI3K/Akt pathway (Figures 5H and S6F). qRT‐PCR results confirmed significantly increased gene expression of *Erk1* and *Erk2*, but not *Mek* expression of BMDMs in LPS and LPS + ABs + anti‐PD1 groups compared with control and LPS + ABs groups (Figure 6A–C). Consistently, western blot analysis showed increased expression of pErk1/2 in the same groups (Figure S7A). Both ABs treatment and Erk inhibitor (PD0325901) treatment significantly deceased the expression of pro‐inflammatory makers CD80 and CD64 in BMDMs with LPS supplementation (Figures 6D,E and S7B–D). In contrast, activation of the Erk pathway by AR234960 increased the expression of these pro‐inflammatory makers in BMDMs with supplementation of LPS and ABs (Figures 6D,E and 7B–D). The Erk inhibitor PD0325901 also decreased the levels of pro‐inflammatory factors IL‐1β, IL‐6, and TNF‐α, while increasing the anti‐inflammatory factor IL‐10 in BMDMs with LPS supplementation (Figure 6F–I). In line with the enhanced pro‐inflammatory state after activation of Erk pathway of ABs treated BMDMs, Erk activator AR234960 increased expression of the pro‐inflammatory factors IL‐1β, IL‐6, and TNF‐α but decreased immunoregulatory factor IL‐10 in BMDMs treated with LPS and ABs (Figure 6F–I).

Genes that control the three rate‐limiting steps of glycolysis including *Hk3*, *Pgam*, and *Ldha* were all downregulated by ABs and PD0325901 in BMDMs treated with LPS (Figure 6J–L). However, activation of the Erk pathway by AR234960 increased the expression of these genes accordingly (Figure 6J–L). To further confirm, the role of the Erk pathway in shifting the metabolism of BMDMs with LPS treatment, seahorse analysis was performed. The results showed that both ABs and PD0325901 shifted ECAR to OXPHOS in BMDMs supplemented with LPS, indicating a shift from glycolysis to OXPHOS (Figures 6M–P and S7E–I). Intriguingly, the activation of Erk pathway by AR234960 increased ECAR and decreased OXPHOS in BMDMs treated with LPS and ABs (Figures 6M–P and S7E–I). These results suggested that ABs reprogramme major metabolic pathways of macrophages via inhibiting Erk pathway downstream of PDL1–PD1 signalling.

### 3.6 ABs promote anti‐inflammatory alveolar macrophages from ALI patients by PDL1–PD1 pathway

We also collected the alveolar macrophages of patients with ALI and found that their alveolar macrophages exhibited pro‐inflammatory phenotype compared with macrophages from peripheral blood of healthy donors (Figure 7A–E). ABs decreased the expression of CD64 and the production of IL‐1β, IL‐6, and TNF‐α in alveolar macrophages of ALI patients (Figure 7A–E). The anti‐inflammation maker CD206 was increased in alveolar macrophages by AB treatment (Figure 7F,G). However, blockade of PDL1–PD1 with anti‐PD1 antibody blunted ABs effects (Figure 7A–G).

To further confirm whether ABs regulate the metabolism of alveolar macrophages of ALI patients, seahorse extracellular flux analysis was performed. The results showed that alveolar macrophages from ALI patients exhibited elevated ECAR, indicating increased glycolysis. However, ABs treatment suppressed the glycolytic activity of alveolar macrophages, leading to a decrease in ECAR. Importantly, when the PDL1–PD1 pathway was blocked with anti‐PD1 antibody, the inhibitory effect of ABs on glycolysis was reversed (Figures 7H,I and S8A,B). Furthermore, ABs treatment induced an elevation in OCR in alveolar macrophages, accompanied by ATP production. However, when the PDL1–PD1 pathway was blocked with anti‐PD1 antibody, the increase in OCR and ATP production induced by ABs was reduced (Figures 7J,K and S8C–E). Collectively, these findings indicate that ABs also inhibit pro‐inflammatory polarization and glycolytic metabolism of alveolar macrophages through PDL1–PD1 pathway in patients with ALI (Figure 7L).

## 4 DISCUSSION

Here, we have outlined a previously unrecognized ability of ABs secreted from apoptotic UC‐MSCs to suppress inflammation and treat ALI. We have also demonstrated that PDL1 is expressed on the membrane of ABs derived from apoptotic UC‐MSCs and interacted with PD1 on macrophages. This interaction leads to a shift in macrophage polarization from a pro‐inflammatory state to an anti‐inflammatory state and reprogrammes the major metabolic pathways of macrophages. Molecular studies showed that PDL1 expressed by ABs made the metabolic switches from glycolysis to OXPHOS of macrophages through the Erk pathway downstream of PDL1–PD1 axis. Importantly, we have confirmed the effects of ABs on metabolic switch of alveolar macrophages from ALI patients, which is dependent on the PDL1–PD1 pathway.

In mammals, impaired clearance of apoptotic cells can lead to exposure of autoantigens, which can trigger autoimmune diseases, such as systemic lupus erythematosus and chronic polyarthritis.^40^, ^41^ Actually, previous research showed that apoptotic cells can exert profound modulatory effects on both the innate and adaptive immune systems.^42^ MSCs have immunomodulatory properties and undergo apoptosis after in vivo application, which contributes to the therapeutic efficacy.^43^, ^44^ Our previous study also found that MSCs disappeared gradually in the injection area of MI mice, accompanied by apoptosis.^13^ According with these results, we found that human UC‐MSCs underwent apoptosis after 24 h intratracheally implantation in ALI mice model. Administration of early‐stage apoptotic cells has been shown to help alleviating disease severity in inflammation models, probably via elicitation of anti‐inflammatory mediators.^45^ Here, we found human UC‐MSCs‐derived ABs could alleviate inflammation of lung tissue, as demonstrated by the decreased inflammatory cell infiltration and decreased expression of inflammatory factors including TNF‐α and IL‐6 in BALF. Inhibition of ABs production from MSCs impaired the therapeutic effect of UC‐MSCs, suggesting the treatment of ALI by human UC‐MSCs relies on the presence of ABs.

Extracellular vesicles (EVs) from viable cells have been extensively studied in recent years, but our knowledge regarding apoptotic vesicles produced by apoptotic cells is still limited. In vivo, macrophages are responsible for the clearance of dying apoptotic cells, both in normal development/tissue homeostasis^46^, ^47^ and at sites of inflammation.^48^ Recent study reported that metabolites released from apoptotic cells, as tissue messengers, which induce anti‐inflammatory effects in macrophages, could alleviate the inflammatory arthritis and lung‐graft rejection.^49^ As to the in vivo fate of ABs, we have found when intratracheally infused, ABs are mainly engulfed by macrophages. EVs derived from viable UC‐MSCs, when primed with interferon γ, have been shown to enhance macrophage phagocytosis and more effectively attenuate *E. coli*‐induced lung injury.^50^ In our study, we have verified that ABs from apoptotic human UC‐MSCs downregulate pro‐inflammatory macrophages in ALI mice. Intriguingly, ABs from MSCs expressed PDL1, which binds to its receptors PD1 on macrophages induced by LPS in this study. Through using neutralizing antibody and siRNA approaches to suppress PDL1–PD1 axis, we have verified that ABs switch macrophages from a pro‐inflammatory state to an anti‐inflammatory state through PDL1–PD1 axis, providing therapeutic benefits in the treatment of ALI and inflammatory arthritis.

PDL1–PD1 axis is now gaining great attention as a potential therapeutic target for the treatment of various inflammatory and autoimmune diseases.^51^ PD1 acts as a metabolic checkpoint in ILC2s, affecting cellular activation and proliferation. Human PD1 agonist ameliorates airway hyper‐reactivity and suppresses lung inflammation.^31^ In our study, the RNA‐seq results revealed that the major metabolic pathway was affected by ABs through PDL1–PD1 axis. PD1 has been reported to modulate T‐cell metabolic reprogramming by inhibiting glycolysis and promoting lipolysis and fatty acid oxidation.^29^ Our results showed that ABs shifted the metabolic pathway from glycolysis to OXPHOS in LPS‐treated BMDMs by seahorse analysis. However, little is known about the mechanism of PDL1/PD1 signalling in macrophage metabolism during inflammation. Evidences have pointed out that Erk signalling pathway is a target of PD1/PDL1 and plays important role in promoting the conversion of energy metabolism to glycolysis by affecting the activity of key metabolic regulators.^52^, ^53^ Previous reports showed that inhibition of Erk can improve the efficacy of anti‐PD1 immunotherapy by downregulating PDL1 expression.^54^ Another study showed PDL1 binding to PD1 promotes M2 macrophage polarization, accompanied by enhanced mitochondrial function and metabolic reprogramming via Erk/Akt/mTOR.^55^ Furthermore, ERK1/2 signalling has been shown to enhance aerobic glycolysis and impair the TCA cycle by promoting the mitochondrial translocation of PGK1, leading to brain tumorigenesis.^56^ ERK1/2 signalling positively controls the function of pro‐inflammatory macrophages by enhancing glycolysis, and its inhibition impairs glucose consumption and lactate production in LPS‐activated macrophages.^57^ In our study, Erk pathway has been confirmed to regulate the expression of three key enzymes in glycolysis, thereby driving the metabolic switch in LPS‐treated BMDMs in response to ABs. Recent studies have also showed that macrophages activated by LPS undergo changes toward glycolysis, whereas macrophages activated with IL‐10 shift OXPHOS, indicating that cell metabolism can direct macrophage polarization state.^21^, ^24^, ^25^, ^58^ However, fatty acid synthesis and oxidation were not changed significantly in LPS‐stimulated macrophages after ABs treatment in our study. These findings pave the way to the better understanding of PDL1–PD1 function in macrophage metabolism and the other metabolic pathway downstream of PDL1–PD1 signalling.

Notably, we replicated and confirmed this immunoregulatory effects of ABs in alveolar macrophages from ALI patients, which indicates the clinical potential of ABs as a therapeutic approach for lung inflammation. ABs decreased the expression of CD64 and increased the expression of CD206 in alveolar macrophages of ALI patients. Moreover, the blockade of the PDL1–PD1 axis attenuated the effects of ABs, suggesting the involvement of this pathway in mediating the immunomodulatory actions of ABs. Metabolic analysis showed that ABs inhibited the pro‐inflammatory polarization and glycolytic metabolism of alveolar macrophages through PDL1–PD1 pathway. Considering the above‐mentioned properties of ABs and its low immunogenicity, our study expands the possibility of applying ABs to the clinical treatment for inflammation such as ALI. Future clinical applications of ABs could involve administration methods like aerosol inhalation or alveolar lavage, expanding the potential for their use in treating inflammation.

## 5 CONCLUSION

In sum, we reveal a key role of ABs in controlling metabolic reprogramming of macrophages via PDL1–PD1 pathway. This metabolic control of ABs can direct the polarization state of macrophages and provide potential therapeutic effect of ALI and other inflammatory diseases. Therapeutic targeting of PDL1–PD1 pathway in macrophages therefore could be beneficial for treatment or prevention of inflammatory diseases associated with ABs.

## AUTHOR CONTRIBUTIONS

Tao Jiang, Bei Li, and Yan Jin designed the experiments, oversaw the collection of results and data interpretation, and drafted the reports. Yanmin Xia, Xiaoning He, and Wenzhe Wang contributed to execution, data acquisition, and interpretation. Jinbo Zhao and Wenhao Liu performed the animal experiments. Shiyu Liu and Songtao Shi contributed to interpretation. The article was prepared by Bei Li, Xiaoning He, Yanmin Xiaa, and Wenzhe Wang. All authors have seen and approved the final version.

## FUNDING INFORMATION

This work was supported by the grants from the National Key Research and Development Program of China (2021YFA1100600 to Bei Li) and the National Natural Science Foundation of China (81991504 to Yan Jin), Shaanxi Provincial Key Research and Development Program (2023‐ZDLSF‐49 to Bei Li and 2021ZDLSF01‐08 to Tao Jiang).

## CONFLICT OF INTEREST STATEMENT

The authors declare no competing interests.

## Supporting information


**Data S1:** Supporting information.

## Data Availability

The datasets used and/or analysed during this study available from the corresponding author upon reasonable request.

## References

[1] Fuchs Y , Steller H . Programmed cell death in animal development and disease. Cell. 2011;147:742‐758. 22078876 10.1016/j.cell.2011.10.033 PMC4511103

[2] Lindsten T , Ross AJ , King A , et al. The combined functions of proapoptotic Bcl‐2 family members bak and bax are essential for normal development of multiple tissues. Mol Cell. 2000;6:1389‐1399. 11163212 10.1016/s1097-2765(00)00136-2 PMC3057227

[3] Arandjelovic S , Ravichandran KS . Phagocytosis of apoptotic cells in homeostasis. Nat Immunol. 2015;16:907‐917. 26287597 10.1038/ni.3253 PMC4826466

[4] Bergmann A , Steller H . Apoptosis, stem cells, and tissue regeneration. Sci Signal. 2010;3:re8. 20978240 10.1126/scisignal.3145re8 PMC2991142

[5] Nagata S . Apoptosis and autoimmune diseases. Ann N Y Acad Sci. 2010;1209:10‐16. 20958310 10.1111/j.1749-6632.2010.05749.x

[6] Cheng J , Zhou T , Liu C , et al. Protection from Fas‐mediated apoptosis by a soluble form of the Fas molecule. Science (New York, NY). 1994;263:1759‐1762. 10.1126/science.7510905 7510905

[7] Sun L , Akiyama K , Zhang H , et al. Mesenchymal stem cell transplantation reverses multiorgan dysfunction in systemic lupus erythematosus mice and humans. Stem Cells. 2009;27:1421‐1432. 19489103 10.1002/stem.68 PMC2704254

[8] Uccelli A , Pistoia V , Moretta L . Mesenchymal stem cells: a new strategy for immunosuppression? Trends Immunol. 2007;28:219‐226. 17400510 10.1016/j.it.2007.03.001

[9] Walter J , Ware LB , Matthay MA . Mesenchymal stem cells: mechanisms of potential therapeutic benefit in ARDS and sepsis. Lancet Respir Med. 2014;2:1016‐1026. 25465643 10.1016/S2213-2600(14)70217-6

[10] Wang G , Cao K , Liu K , et al. Kynurenic acid, an IDO metabolite, controls TSG‐6‐mediated immunosuppression of human mesenchymal stem cells. Cell Death Differ. 2018;25:1209‐1223. 29238069 10.1038/s41418-017-0006-2 PMC6030103

[11] Poon IK , Lucas CD , Rossi AG , Ravichandran KS . Apoptotic cell clearance: basic biology and therapeutic potential. Nat Rev Immunol. 2014;14:166‐180. 24481336 10.1038/nri3607 PMC4040260

[12] Liu D , Kou X , Chen C , et al. Circulating apoptotic bodies maintain mesenchymal stem cell homeostasis and ameliorate osteopenia via transferring multiple cellular factors. Cell Res. 2018;28:918‐933. 30030518 10.1038/s41422-018-0070-2 PMC6123409

[13] Liu H , Liu S , Qiu X , et al. Donor MSCs release apoptotic bodies to improve myocardial infarction via autophagy regulation in recipient cells. Autophagy. 2020;16:2140‐2155. 31959090 10.1080/15548627.2020.1717128 PMC7751634

[14] Zheng C , Sui B , Zhang X , et al. Apoptotic vesicles restore liver macrophage homeostasis to counteract type 2 diabetes. J Extracell Vesicles. 2021;10:e12109. 34084287 10.1002/jev2.12109 PMC8144839

[15] Bhattacharya J , Matthay MA . Regulation and repair of the alveolar‐capillary barrier in acute lung injury. Annu Rev Physiol. 2013;75:593‐615. 23398155 10.1146/annurev-physiol-030212-183756

[16] Divangahi M , King IL , Pernet E . Alveolar macrophages and type I IFN in airway homeostasis and immunity. Trends Immunol. 2015;36:307‐314. 25843635 10.1016/j.it.2015.03.005

[17] Kopf M , Schneider C , Nobs SP . The development and function of lung‐resident macrophages and dendritic cells. Nat Immunol. 2015;16:36‐44. 25521683 10.1038/ni.3052

[18] Lee H , Zhang D , Wu J , Otterbein LE , Jin Y . Lung epithelial cell‐derived microvesicles regulate macrophage migration via MicroRNA‐17/221‐induced integrin beta1 recycling. J Immunol. 2017;199:1453‐1464. 28674181 10.4049/jimmunol.1700165 PMC5561736

[19] Weavers H , Evans IR , Martin P , Wood W . Corpse engulfment generates a molecular memory that primes the macrophage inflammatory response. Cell. 2016;165:1658‐1671. 27212238 10.1016/j.cell.2016.04.049 PMC4912690

[20] Tannahill GM , Curtis AM , Adamik J , et al. Succinate is an inflammatory signal that induces IL‐1beta through HIF‐1alpha. Nature. 2013;496:238‐242. 23535595 10.1038/nature11986 PMC4031686

[21] Rodriguez‐Prados JC , Traves PG , Cuenca J , et al. Substrate fate in activated macrophages: a comparison between innate, classic, and alternative activation. J Immunol. 2010;185:605‐614. 20498354 10.4049/jimmunol.0901698

[22] Lampropoulou V , Sergushichev A , Bambouskova M , et al. Itaconate links inhibition of succinate dehydrogenase with macrophage metabolic remodeling and regulation of inflammation. Cell Metab. 2016;24:158‐166. 27374498 10.1016/j.cmet.2016.06.004 PMC5108454

[23] Mills EL , Kelly B , Logan A , et al. Succinate dehydrogenase supports metabolic repurposing of mitochondria to drive inflammatory macrophages. Cell. 2016;167:457‐470.e13. 27667687 10.1016/j.cell.2016.08.064 PMC5863951

[24] Jha AK , Huang SC , Sergushichev A , et al. Network integration of parallel metabolic and transcriptional data reveals metabolic modules that regulate macrophage polarization. Immunity. 2015;42:419‐430. 25786174 10.1016/j.immuni.2015.02.005

[25] Ip WKE , Hoshi N , Shouval DS , Snapper S , Medzhitov R . Anti‐inflammatory effect of IL‐10 mediated by metabolic reprogramming of macrophages. Science. 2017;356:513‐519. 28473584 10.1126/science.aal3535 PMC6260791

[26] Riella LV , Paterson AM , Sharpe AH , Chandraker A . Role of the PD‐1 pathway in the immune response. Am J Transplant. 2012;12:2575‐2587. 22900886 10.1111/j.1600-6143.2012.04224.x PMC3784243

[27] Ni K , Liu M , Zheng J , et al. PD‐1/PD‐L1 pathway mediates the alleviation of pulmonary fibrosis by human mesenchymal stem cells in humanized mice. Am J Respir Cell Mol Biol. 2018;58:684‐695. 29220578 10.1165/rcmb.2017-0326OC

[28] Su D , Tsai HI , Xu Z , et al. Exosomal PD‐L1 functions as an immunosuppressant to promote wound healing. J Extracell Vesicles. 2019;9:1709262. 33133428 10.1080/20013078.2019.1709262 PMC7580831

[29] Patsoukis N , Bardhan K , Chatterjee P , et al. PD‐1 alters T‐cell metabolic reprogramming by inhibiting glycolysis and promoting lipolysis and fatty acid oxidation. Nat Commun. 2015;6:6692. 25809635 10.1038/ncomms7692 PMC4389235

[30] Bengsch B , Johnson AL , Kurachi M , et al. Bioenergetic insufficiencies due to metabolic alterations regulated by the inhibitory receptor PD‐1 are an early driver of CD8(+) T cell exhaustion. Immunity. 2016;45:358‐373. 27496729 10.1016/j.immuni.2016.07.008 PMC4988919

[31] Helou DG , Shafiei‐Jahani P , Lo R , et al. PD‐1 pathway regulates ILC2 metabolism and PD‐1 agonist treatment ameliorates airway hyperreactivity. Nat Commun. 2020;11:3998. 32778730 10.1038/s41467-020-17813-1 PMC7417739

[32] Mikawa K , Nishina K , Takao Y , Obara H . ONO‐1714, a nitric oxide synthase inhibitor, attenuates endotoxin‐induced acute lung injury in mouse. Anesth Analg. 2003;97:1751‐1755. 14633554 10.1213/01.ANE.0000086896.90343.13

[33] Zhai Q , Dong J , Zhang X , et al. Mesenchymal stem cells enhance therapeutic effect and prevent adverse gastrointestinal reaction of methotrexate treatment in collagen‐induced arthritis. Stem Cells Int. 2021;2021:8850820‐8850812. 33505476 10.1155/2021/8850820 PMC7814936

[34] Love MI , Huber W , Anders S . Moderated estimation of fold change and dispersion for RNA‐seq data with DESeq2. Genome Biol. 2014;15:550. 25516281 10.1186/s13059-014-0550-8 PMC4302049

[35] Yanez‐Mo M , Siljander PR , Andreu Z , et al. Biological properties of extracellular vesicles and their physiological functions. J Extracell Vesicles. 2015;4:27066. 25979354 10.3402/jev.v4.27066 PMC4433489

[36] Morrison TJ , Jackson MV , Cunningham EK , et al. Mesenchymal stromal cells modulate macrophages in clinically relevant lung injury models by extracellular vesicle mitochondrial transfer. Am J Respir Crit Care Med. 2017;196:1275‐1286. 28598224 10.1164/rccm.201701-0170OC PMC5694830

[37] Liesa M , Shirihai OS . Mitochondrial dynamics in the regulation of nutrient utilization and energy expenditure. Cell Metab. 2013;17:491‐506. 23562075 10.1016/j.cmet.2013.03.002 PMC5967396

[38] Wieman HL , Wofford JA , Rathmell JC . Cytokine stimulation promotes glucose uptake via phosphatidylinositol‐3 kinase/Akt regulation of Glut1 activity and trafficking. Mol Biol Cell. 2007;18:1437‐1446. 17301289 10.1091/mbc.E06-07-0593 PMC1838986

[39] Marko AJ , Miller RA , Kelman A , Frauwirth KA . Induction of glucose metabolism in stimulated T lymphocytes is regulated by mitogen‐activated protein kinase signaling. PloS One. 2010;5:e15425. 21085672 10.1371/journal.pone.0015425 PMC2978105

[40] Franz S , Gaipl US , Munoz LE , et al. Apoptosis and autoimmunity: when apoptotic cells break their silence. Curr Rheumatol Rep. 2006;8:245‐247. 16839503 10.1007/s11926-006-0001-y

[41] Kawane K , Ohtani M , Miwa K , et al. Chronic polyarthritis caused by mammalian DNA that escapes from degradation in macrophages. Nature. 2006;443:998‐1002. 17066036 10.1038/nature05245

[42] Perruche S , Zhang P , Liu Y , Saas P , Bluestone JA , Chen W . CD3‐specific antibody‐induced immune tolerance involves transforming growth factor‐beta from phagocytes digesting apoptotic T cells. Nat Med. 2008;14:528‐535. 18438416 10.1038/nm1749

[43] Galleu A , Riffo‐Vasquez Y , Trento C , et al. Apoptosis in mesenchymal stromal cells induces in vivo recipient‐mediated immunomodulation. Sci Transl Med. 2017;9:eaam7828. 29141887 10.1126/scitranslmed.aam7828

[44] Weiss DJ , English K , Krasnodembskaya A , Isaza‐Correa JM , Hawthorne IJ , Mahon BP . The Necrobiology of mesenchymal stromal cells affects therapeutic efficacy. Front Immunol. 2019;10:1228. 31214185 10.3389/fimmu.2019.01228 PMC6557974

[45] Gatza E , Rogers CE , Clouthier SG , et al. Extracorporeal photopheresis reverses experimental graft‐versus‐host disease through regulatory T cells. Blood. 2008;112:1515‐1521. 18411417 10.1182/blood-2007-11-125542 PMC2515148

[46] Jacobson MD , Weil M , Raff MC . Programmed cell death in animal development. Cell. 1997;88:347‐354. 9039261 10.1016/s0092-8674(00)81873-5

[47] Wood W , Turmaine M , Weber R , et al. Mesenchymal cells engulf and clear apoptotic footplate cells in macrophageless PU.1 null mouse embryos. Development. 2000;127:5245‐5252. 11076747 10.1242/dev.127.24.5245

[48] Martin P , Leibovich SJ . Inflammatory cells during wound repair: the good, the bad and the ugly. Trends Cell Biol. 2005;15:599‐607. 16202600 10.1016/j.tcb.2005.09.002

[49] Medina CB , Mehrotra P , Arandjelovic S , et al. Metabolites released from apoptotic cells act as tissue messengers. Nature. 2020;580:130‐135. 32238926 10.1038/s41586-020-2121-3 PMC7217709

[50] Varkouhi AK , Jerkic M , Ormesher L , et al. Extracellular vesicles from interferon‐gamma‐primed human umbilical cord mesenchymal stromal cells reduce Escherichia coli‐induced acute lung injury in rats. Anesthesiology. 2019;130:778‐790. 30870158 10.1097/ALN.0000000000002655

[51] Qin W , Hu L , Zhang X , et al. The diverse function of PD‐1/PD‐L pathway beyond cancer. Front Immunol. 2019;10:2298. 31636634 10.3389/fimmu.2019.02298 PMC6787287

[52] Papa S , Choy PM , Bubici C . The ERK and JNK pathways in the regulation of metabolic reprogramming. Oncogene. 2019;38(13):2223‐2240. 30487597 10.1038/s41388-018-0582-8 PMC6398583

[53] Piao W , Li L , Saxena V , et al. PD‐L1 signaling selectively regulates T cell lymphatic transendothelial migration. Nat Commun. 2022;13(1):2176. 35449134 10.1038/s41467-022-29930-0 PMC9023578

[54] Luo M , Xia Y , Wang F , et al. PD0325901, an ERK inhibitor, enhances the efficacy of PD‐1 inhibitor in non‐small cell lung carcinoma. Acta Pharm Sin B. 2021;11(10):3120‐3133. 34729305 10.1016/j.apsb.2021.03.010 PMC8546891

[55] Wei Y , Liang M , Xiong L , Su N , Gao X , Jiang Z . PD‐L1 induces macrophage polarization toward the M2 phenotype via Erk/Akt/mTOR. Exp Cell Res. 2021;402(2):112575. 33771483 10.1016/j.yexcr.2021.112575

[56] Li X , Jiang Y , Meisenhelder J , et al. Mitochondria‐translocated PGK1 functions as a protein kinase to coordinate glycolysis and the TCA cycle in tumorigenesis. Mol Cell. 2016;61:705‐719. 26942675 10.1016/j.molcel.2016.02.009 PMC4888784

[57] Través PG , de Atauri P , Marín S , et al. Relevance of the MEK/ERK signaling pathway in the metabolism of activated macrophages: a metabolomic approach. J Immunol. 2012;188:1402‐1410. 22190182 10.4049/jimmunol.1101781

[58] Pearce EL , Pearce EJ . Metabolic pathways in immune cell activation and quiescence. Immunity. 2013;38:633‐643. 23601682 10.1016/j.immuni.2013.04.005 PMC3654249

