# Supplementary material for: Apoptotic bodies inhibit inflammation by PDL1–PD1‐mediated macrophage metabolic reprogramming
Source: Cell Prolif. 2023 Aug 8;57(1):e13531. doi: 10.1111/cpr.13531 (PMC10771117; doi:10.1111/cpr.13531)
Supplement: Supplementary file 1 — Data S1: Supporting information. [file CPR-57-e13531-s001.docx]

**SUPPLEMENTARY INFORMATION**

Supplementary information includes nine supplementary figures (Figure S1-S9), one table and experimental procedures.

## Supplementary Figures


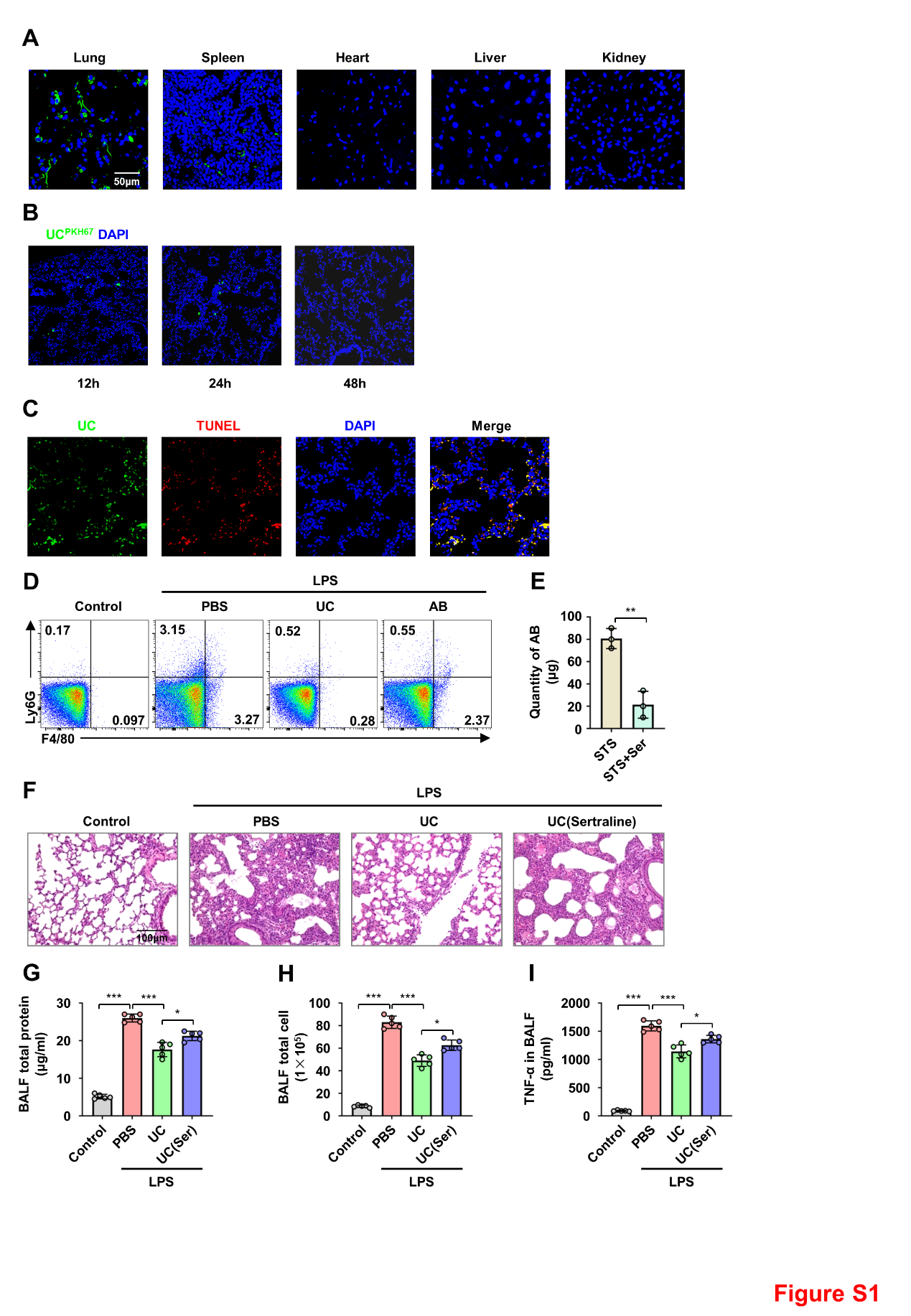


**Figure S1. Apoptotic Bodies Released by UC-MSCs Prevent LPS-Induced Inflammatory Lung Injury.** (A) Confocal microscopy images showed that PKH67 labelled UC-MSCs were mainly found in lung tissue after 24 h transplantation. (B) Confocal microscopy images showed that decreased UC-MSCs in lung tissue at 12 h, 24 h and 48 h after the injection. (C) TUNEL staining for lung tissue after 24 h transplantation. UC-MSCs were pre-labeled with PKH67 (green), and apoptotic nuclei were indicated by red fluorescence. Scale bar, 50 μm. (D) Flow cytometry detection of neutrophils (Ly6G) and macrophages (F4/80) in BAL fluid of ALI mice treated with UC-MSCs and ABs. (E) Quantity of ABs released by UC-MSCs was decreased with treatment of sertraline (n=3). (F) H&E staining showed inhibition of ABs release compromised the effect of UC-MSCs on the alleviation of lung inflammation. Scale bar, 100 μm. (G) Total protein in BAL fluid was measured by BCA assay (n=5). (H) Treatment with UC-MSCs significantly attenuated cell infiltration of lung and the therapeutic effect declined when the apoptosis of UC-MSCs was inhibited by sertraline (n=5). (I) ELISA for TNFα in BAL fluid (n=5).


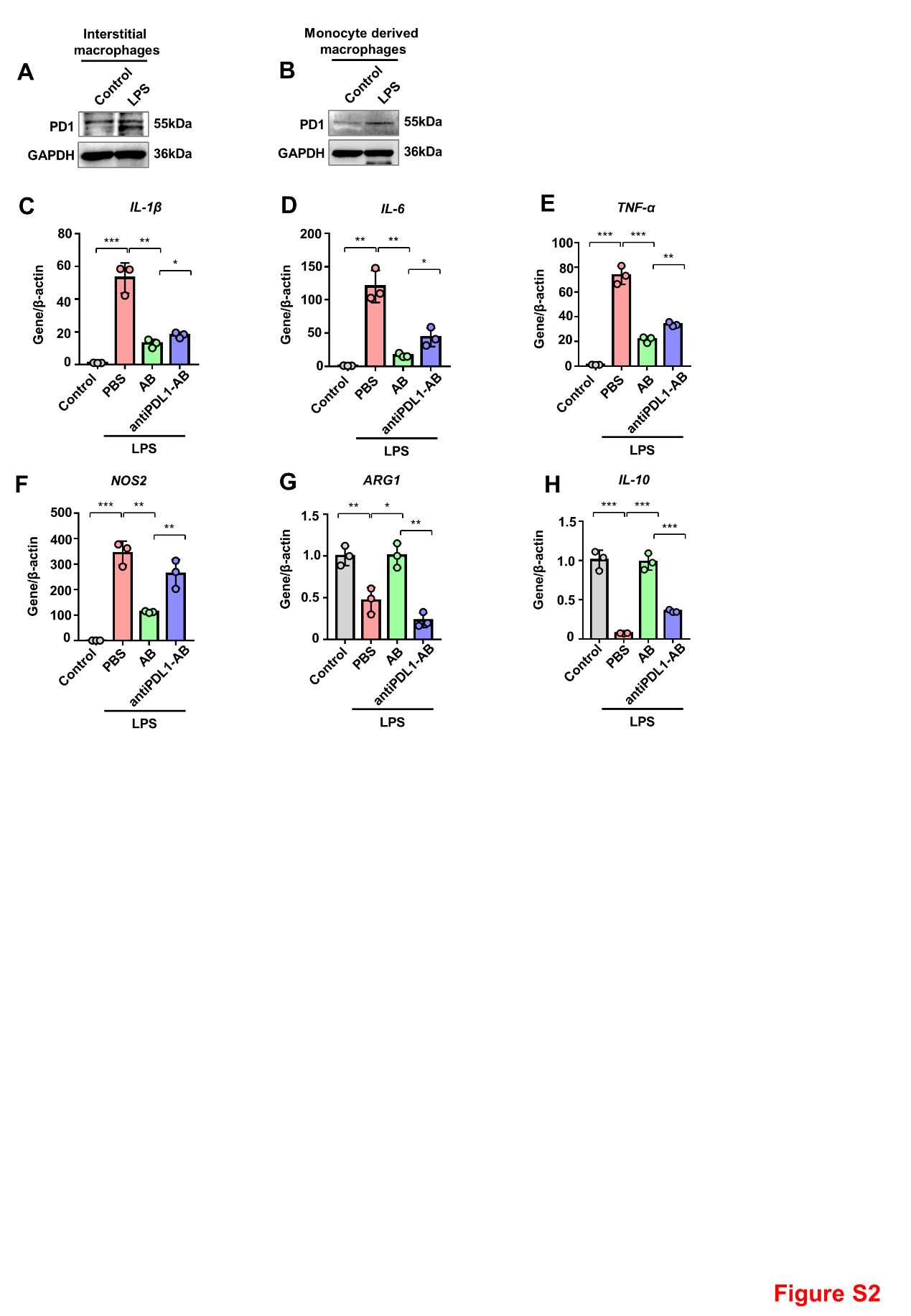


**Figure S2. ABs inhibit proinflammation of macrophages through PDL1-PD1 pathway.** (A and B) The expressions of PD1 on the interstitial macrophages (A) and monocyte derived macrophages (B) of mice were increased after stimulated by LPS. (C-H) The expression levels of the genes related to pro-inflammatory cytokines (IL-1β (C), IL-6 (D), TNF-α (E) and NOS2 (F)) and anti-inflammatory cytokines (Arg1 (G) and IL-10 (H)) of macrophages were detected by qRT-PCR analysis (n=3).

**
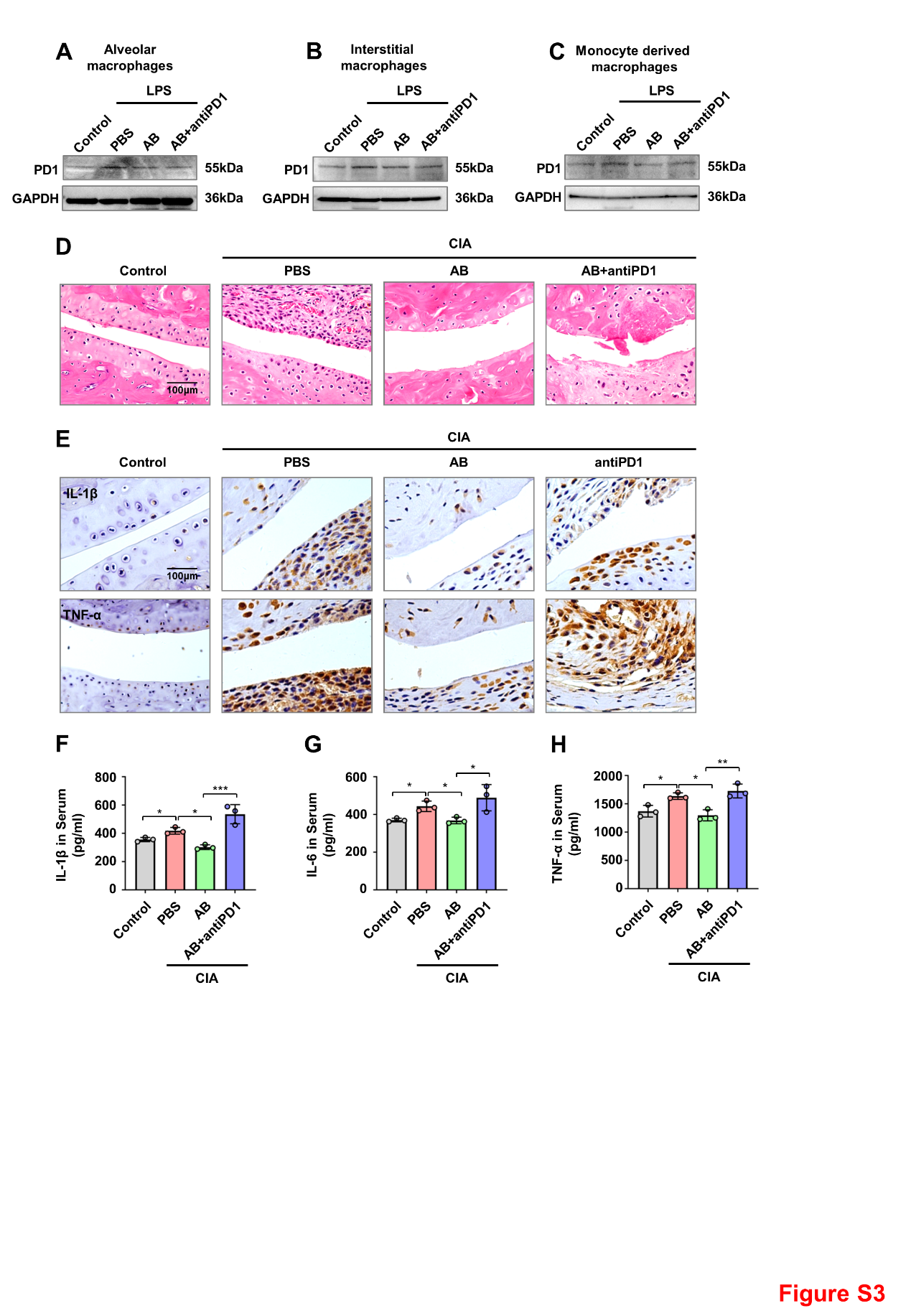
**

**Figure S3. Blockage of PDL1-PD1 by Anti-PD1 Antibody Attenuated the Therapeutic Effect of ABs on Type-Ⅱ Collagen Induced Inflammatory Arthritis.** (A-C) The expressions of PD1 on the alveolar macrophages (A), interstitial macrophages (B) and monocyte derived macrophages (C) of mice were decreased after treatment with ABs and anti-PD1 antibody. (D) H&E staining of paraffin sections of hind paws of CIA mice. Scale bar, 100 μm. (E) Representative images of immunohistochemical staining for IL-1β and TNF-α in joints of CIA mice. Scale bar, 100 μm. (F-H) ELISA for IL-1β (F), IL-6 (G), TNFα (H) in serum of CIA mice. Group size n=5 mice per group. *P<0.05; **P< 0.01; ***P< 0.001. Error bars are mean ± SD.


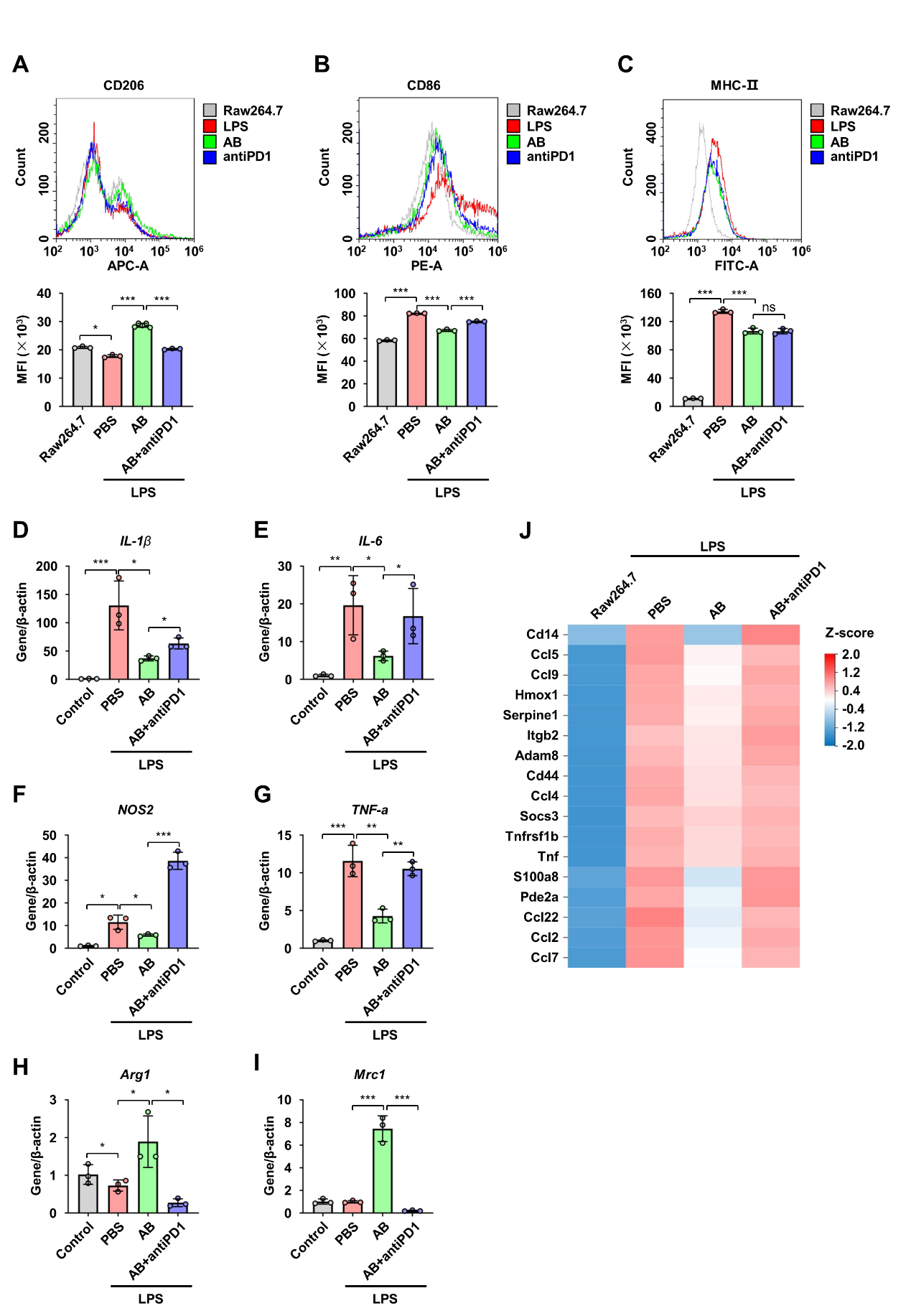


**Figure S4. Blockade of PDL1-PD1 Attenuates the Effect of ABs on Promoting Anti-Inflammatory Polarization of Macrophage.** (A-C) Representative flow cytometry histogram and quantification of CD206 (A), CD86 (B) and MHC-Ⅱ (C) expression in Raw264.7 and Raw264.7 treated with LPS, ABs or ABs+anti-PD1 (n=3). (D-I) qRT-PCR analysis of IL-1β (D), IL-6 (E), NOS2 (F), TNF-α (G), Arg1 (H) and Mrc1 (I) in BMDMs and BMDMs treated with LPS, ABs or ABs+anti-PD1 (n=3). (J) Heatmap depicts differential expression levels for proinflammatory cytokine and chemokine transcripts in Raw264.7 and Raw264.7 treated with LPS, ABs or ABs+anti-PD1. *P<0.05; **P< 0.01; ***P< 0.001. Error bars are mean ± SD.


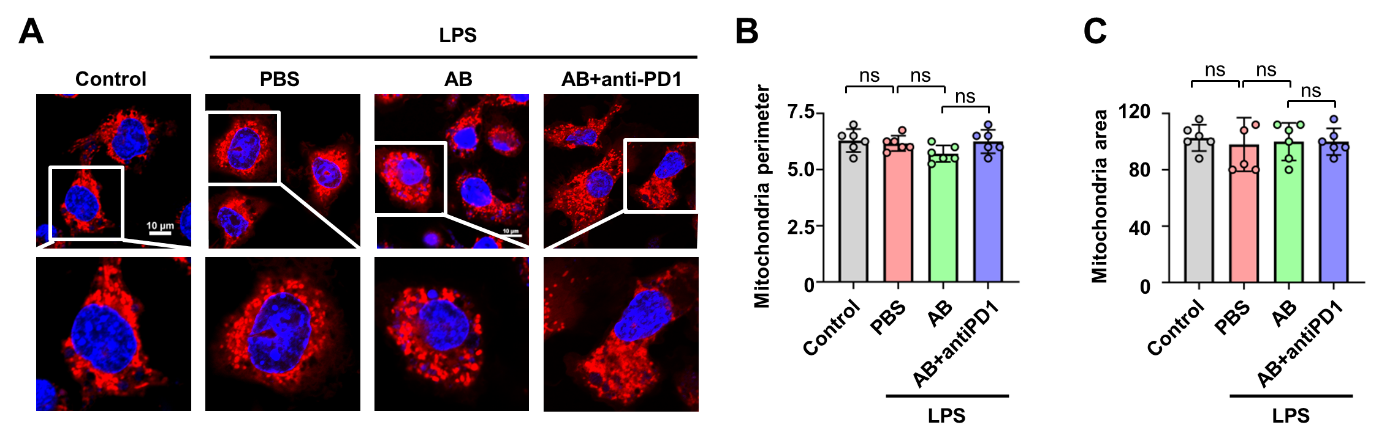


**Figure S5. No Effects on Mitochondrial Networks of blockade of** **PDL1-PD1 pathway in BMDMs.** (A) Confocal microscopy images showing no significant changes of mitochondrial networks in BMDMs with supplementation of LPS, LPS+ABs or LPS+ABs+anti-PD1. Scale bars, 10 μm. (B) Quantification of mitochondria perimeter (n=6). (C) Quantification of mitochondria area (n=6).


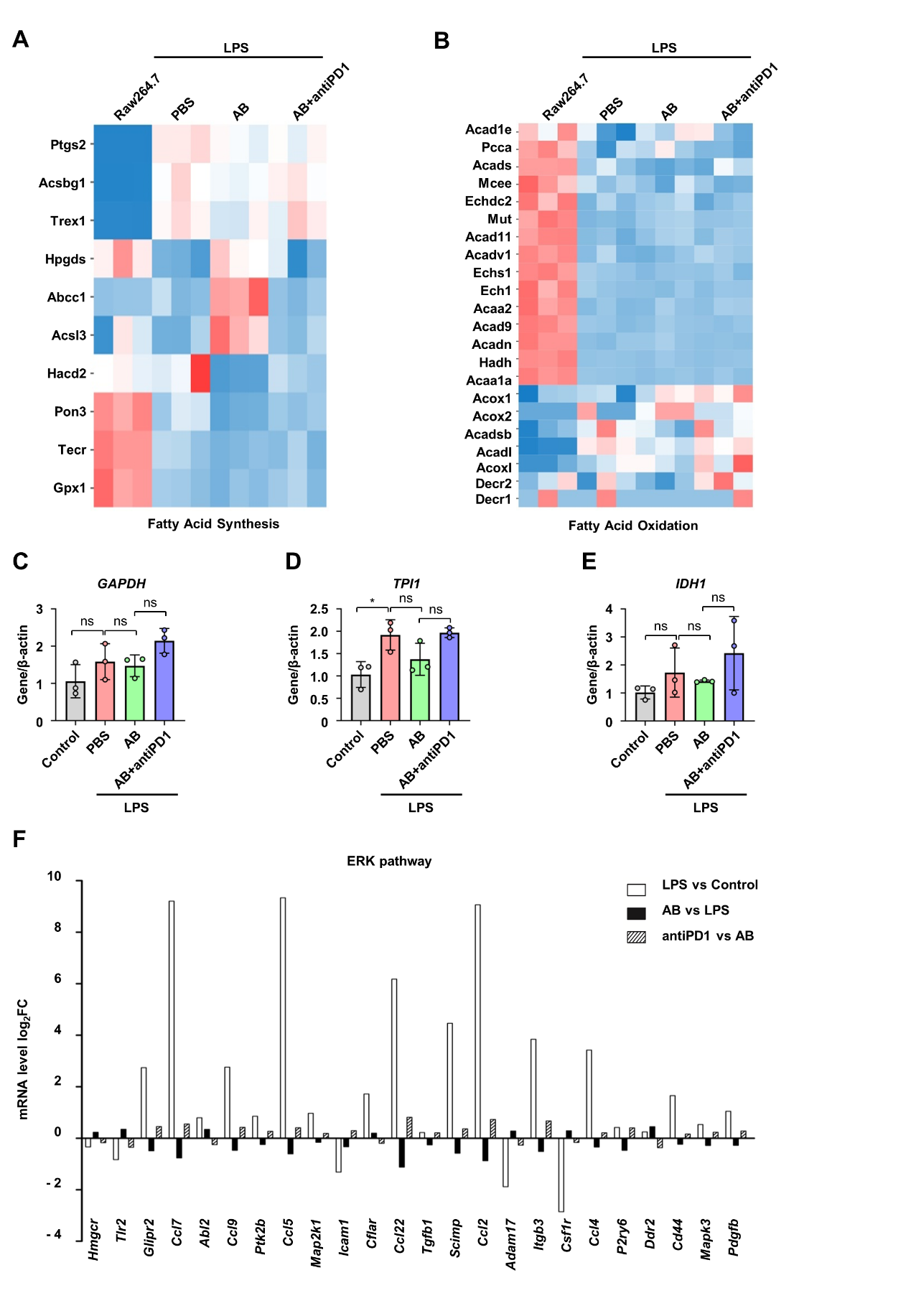


**Figure S6. Differential Gene Expression in Metabolic Pathways in RAW264.7.** (A) Heatmap depicts differential expression levels for fatty acid synthesis in Raw264.7 and Raw264.7 treated with LPS, ABs or ABs+anti-PD1. (B) Heatmap depicts differential expression levels for fatty acid oxidation in Raw264.7 and Raw264.7 treated with LPS, ABs or ABs+anti-PD1. (C-E) qRT-PCR analysis of GAPDH (C), TPI1 (D) and IDH1 (E) in BMDMs with supplementation of LPS, LPS+ABs or LPS+ABs+anti-PD1 (n=3). (F) ERK pathway analysis was performed on the unique gene IDs exhibiting any altered isoforms.


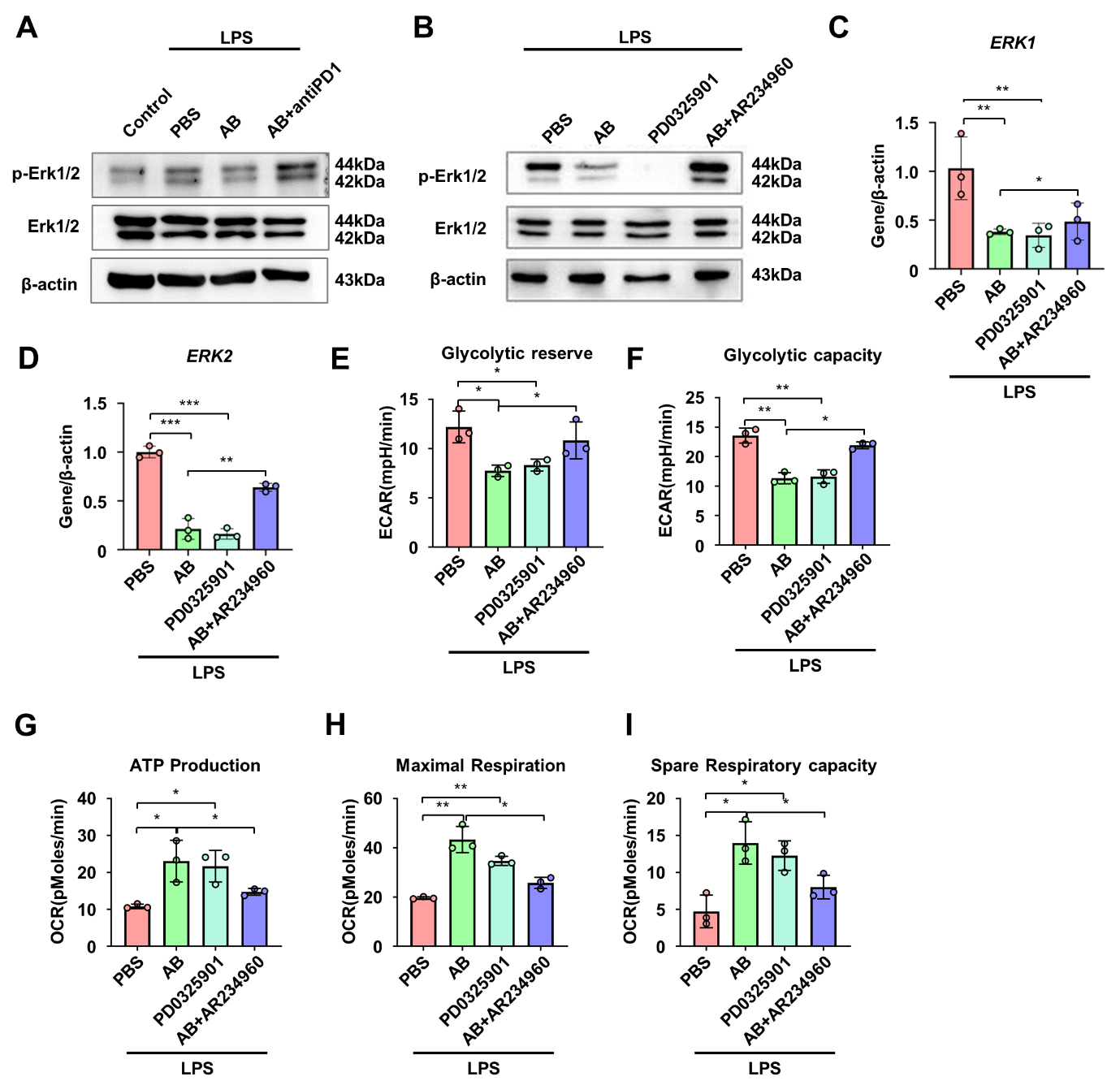


**Figure S7. ABs Reprogram Metabolic Pathways of Macrophages through Erk.** (A) The protein expression of Erk1/2 and pErk1/2 in BMDMs and BMDMs with supplementation of LPS, LPS+ABs or LPS+ABs+anti-PD1. (B) The protein expression of Erk1/2 and pErk1/2 in BMDMs with supplementation of LPS, LPS+ABs, LPS+ABs+PD0325901 or LPS+ABs+AR234960. (C and D) qRT-PCR analysis of ERK1 (C), ERK2 (D) in LPS induced BMDMs with supplementation of ABs or PD0325901 or ABs+AR234960 (n=3). (E and F) Effects of ERK pathway on glycolytic reserve (E) and glycolytic capacity (F) in ECAR assay. (G-I) Effects of ERK pathway on ATP production (G), maximal respiration (H) and spare respiratory capacity (I) in OCR assay (n=3). *P<0.05; **P< 0.01; ***P< 0.001. Error bars are mean ± SD.


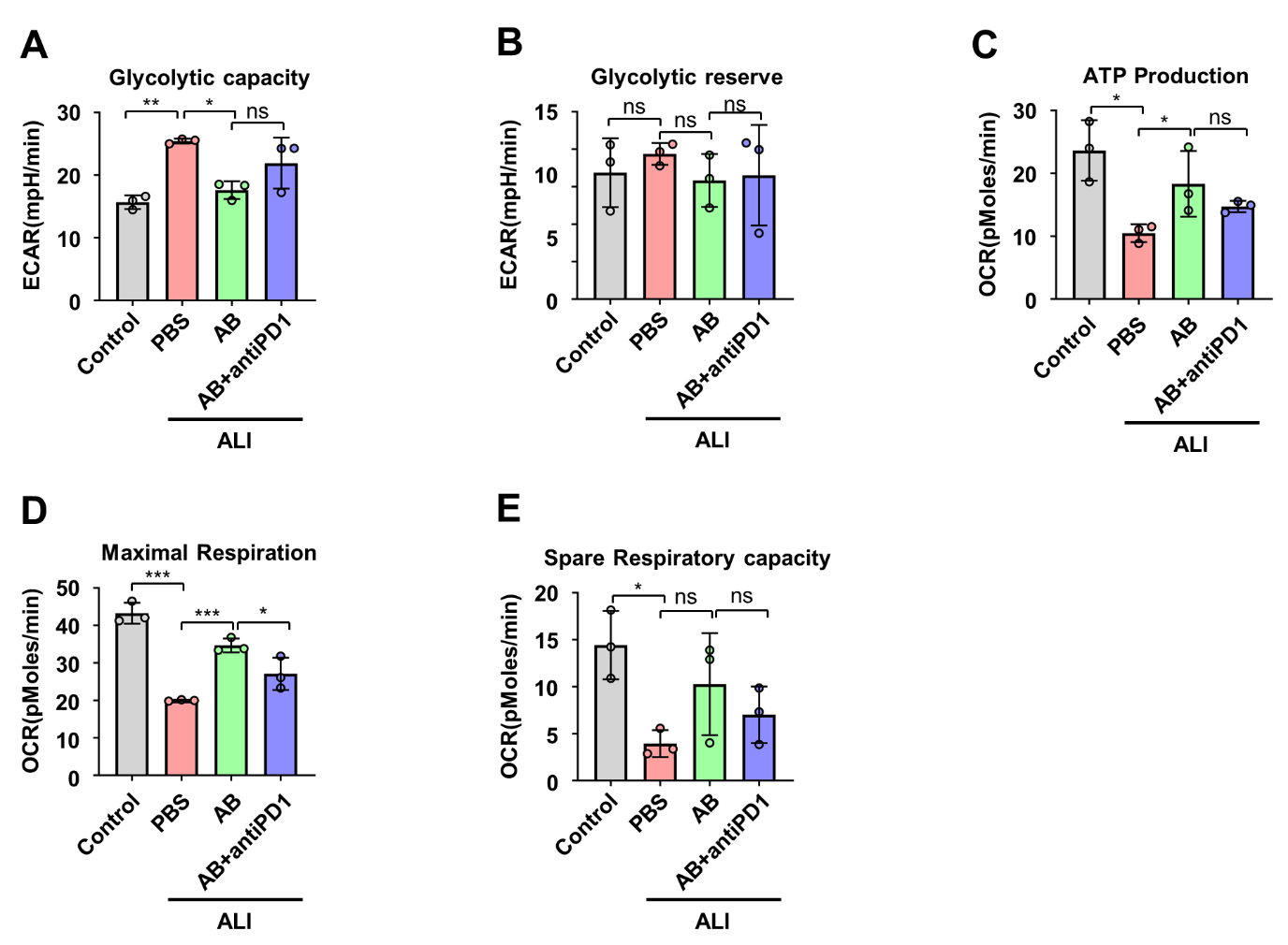


**Figure S8. ABs Promote Metabolic Reprogramming of Alveolar Macrophages from ALI Patients through PD1-PDL1 pathway.** (A and B) glycolytic capacity (A) and glycolytic reserve (B) in ECAR assay (n=3). (C-E) ATP production (C), maximal respiration (D) and spare respiratory capacity (E) in OCR assay (n=3). ns, not significant. *P<0.05; **P< 0.01; ***P< 0.001. Error bars are mean ± SD.

**
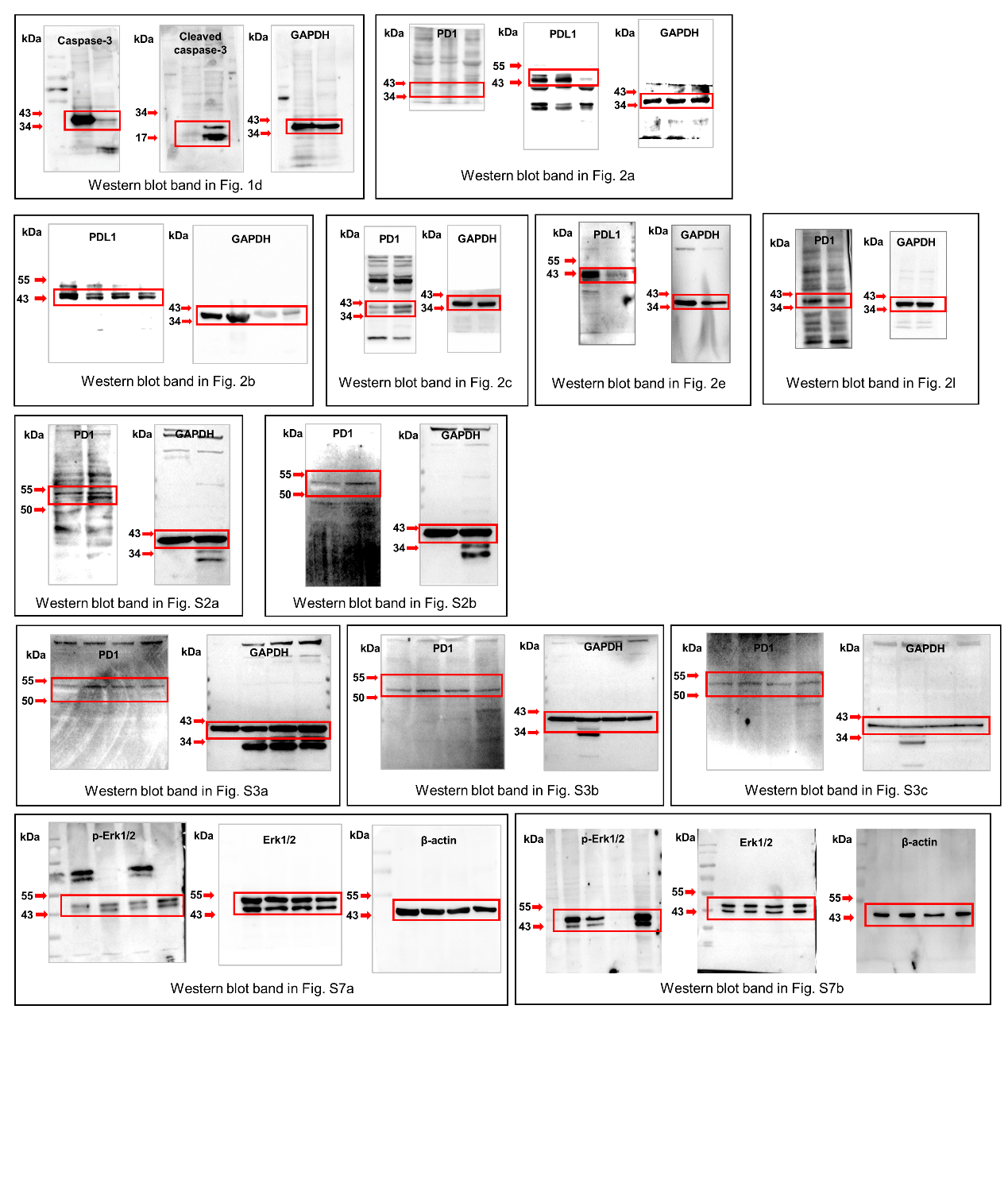
**

**Figure S9.** Uncropped images of the western blot bands.

## Supplementary Table

**Sequence of primers for qRT-PCR.**

| Gene |  | Sequences (5’-3’) |
| --- | --- | --- |
| m-β-actin | Forward | CATCCGTAAAGACCTCTATGCCAAC |
|  | Reverse | ATGGAGCCACCGATCCACA |
| m-PD1 | Forward | TAGTGGGTATCCCTGTATTGCT |
|  | Reverse | CTCCTCCTTCAGAGTGTCGTC |
| m-IL-1β | Forward | CATCAGCACCTCACAAGCAGA |
| m-IL-6  m-TNF-a  m-NOS2  m-Arg-1 | Reverse  Forward  Reverse  Forward  Reverse  Forward  Reverse  Forward  Reverse | TGGGGAAGGCATTAGAAACAG  GAGCCCACCAAGAACGATAGTC  TTTCTCATTTCCACGATTTCCC  AGTCAACCTCCTCTCTGCCG  GCAATGACTCCAAAGTAGACCTG  TGGAGCGAGTTGTGGATTGTC  GTGAGGGCTTGGCTGAGTGA  TCCACCCCCCTTCCCTCCCG  CCTGCCACCCGTAGTTGCCTCG |
| m-Mrc1 | Forward | CTGGTGAACGGAATGATTGTGTAGT |
|  | Reverse | CTTTAGAAGAGCCCTTGGGTTGA |
| m-HK3 | Forward | GAGGGCGAGTGTTTGAACGAC |
|  | Reverse | CCACCACCATCCACAGAGGG |
| m-PGAM  m-LDHA  m-GAPDH  m-TPI1  m-IDH1  m-ERK1 | Forward  Reverse  Forward  Reverse  Forward  Reverse  Forward  Reverse  Forward  Reverse  Forward | GTTGCGAGATGCTGGCTATGAAT  GAGGCGGTGGGACATCATAAGA  GACAAGGAGCAGTGGAAGGAGG  CTTAATCATGGTGGAAATGGGATG  AGGTCGGTGTGAACGGATTTG  GGGGTCGTTGATGGCAACA  ACTGGAAGATGAACGGGAGGAA  TGGCAAAGTCGATGTAAGCGG  GTCAGACTCAGTCGCCCAAGGTT  TCCTTAGTCATAAAGCCAGCCTCAA  CAAGGAGCGGCTGAAGGAGT |
|  | Reverse | AGGGGATTGGAGTGGGAGAA |
| m-ERK2  m-MEK  h-GAPDH  h-PDL1 | Forward  Reverse  Forward  Reverse  Forward  Reverse  Forward | AAGTCCATTGATATTTGGTCTGTGG  AGCCTGTTCAACTTCAATCCTCTT  GTGTTTGAAATCCCCTCCCC  GTCACTCTCCTCCGTCCCG  GGAGCGAGATCCCTCCAAAAT  GGCTGTTGTCATACTTCTCATGG  GAACTACCTCTGGCACATCCTC |
|  | Reverse | CCACATTTTTTCACATCCATCAT |

## Supplementary Experimental Procedures

**Animal Models of Inflammatory Arthritis.** The collagen induced arthritis (CIA) mouse model was established as previously described [1]. Briefly, female DBA/1 mice (6-8 weeks old) were obtained from Beijing Vital River Laboratory Animal Technology Co., Ltd., 100 μg bovine type II collagen (CII) (Chondrex, Redmond, WA, USA) was emulsified with an equal volume of Freund’s complete adjuvant. Mice were injected at the base of tail with 50 μl emulsifier. On day 21, the mice received a booster injection of collagen emulsion in Freund’s incomplete adjuvant. CIA mice were injected ABs after anti-PD1 treatment for 24 hours. Mice were sacrificed after AB or ABs+anti-PD1 treatment for 4 weeks. Serum was harvest and the level of IL-1β, IL-6 and TNF-α were measured the level of IL-1β, IL-6 and TNF-α using ELISA kit.

**Histological analysis.** For histological analysis, lungs were fixed in 4% paraformaldehyde, hind limbs were fixed and decalcified in 17% EDTA, embedded in paraffin, and 4 μm thick serial sections were performed for histological and immunohistochemical staining.

**RNA Isolation and Real-time Quantitative PCR Analysis.** Total RNA was extracted from the cells in varied states using TRIZOL reagent (Invitrogen, Carlsbad, CA, USA) according to the manufacturer’s protocol. Then the reverse transcription was conducted for 1μg RNA with a PrimeScript RT reagent kit (TaKaRa, Japan). All real time PCR reactions were performed on Bio-Rad CFX Manager (Bio-Rad Laboratories Inc., Hercules, CA). Relative quantization of mRNA abundance for the genes of interest were calculated using the 2^ˉ△△Ct^ method. All assays were done with three wells per condition in over three independent experiments. β-actin was used to standardize the mRNA transcription levels. The primer sequences are listed in supplementary table.

**Western Blot.** The cells were collected and lysed in radioimmunoprecipitation assay (RIPA) buffer. After centrifugation at 12,000 × g for 5 min, the lysates were quantified with BCA. Samples (20 μg) were separated by 10% or 15% gradient sodium dodecyl sulfate-polyacrylamide gel electrophoresis (SDS-PAGE), and proteins were transferred to PVDF membranes. After incubated in blocking solution (TBS containing 0.05% Tween-20 and 5% non-fat dry milk) for 1 h at room temperature, the membranes were reacted with primary antibodies at 4°C overnight. Antibodies to mouse phospho-Erk1/2 (Thr202/Tyr204), Erk1/2, caspase3, cleaved caspase3 were purchased from Abcam (Cambridge, UK). PD1, PDL1, MFN1, MFN2 were purchased from Cell Signalling Technology (CST, Danvers, MA, USA). Antibodies to mouse GAPDH and β-actin was purchased from Boster (Wuhan, China). The membranes were washed and exposed to secondary antibodies (1/40,000 dilution) for 1 h at room temperature. The bound antibodies were visualized using ECL blotting detection reagents (Amersham Biosciences, Piscataway, NJ, USA). The band intensity was quantified by NIH ImageJ software.

## Supplementary Reference

[1] Zhang Q, Li Q, Zhu J, Guo H, Zhai Q, Li B, et al. Comparison of therapeutic effects of different mesenchymal stem cells on rheumatoid arthritis in mice. Peer J. 2019; 7: e7023.
